# Supplementary material for: A machine learning approach for identifying anatomical biomarkers of early mild cognitive impairment
Source: PeerJ. 2024 Dec 13;12:e18490. doi: 10.7717/peerj.18490 (PMC11648692; doi:10.7717/peerj.18490)
Supplement: Supplemental Information 2 [file peerj-12-18490-s002.docx]

**Supplementary Material**

**A Machine Learning Approach for Identifying Anatomical Biomarkers of Early Mild Cognitive Impairment**

Alwani Liyana Ahmad^1,2,3^, Jose Sanchez-Bornot^4^, Roberto C. Sotero^5^, Damien Coyle^6^, Zamzuri Idris^2,3,7^, Ibrahima Faye^1,8, *^, for the Alzheimer’s Disease Neuroimaging Initiative^©^

^1^ Department of Fundamental and Applied Sciences, Faculty of Science and Information Technology, Universiti Teknologi PETRONAS, Perak, Malaysia.

^2^ Department of Neurosciences, Hospital Universiti Sains Malaysia, Kelantan, Malaysia.

^3^ Brain and Behaviour Cluster, School of Medical Sciences, Universiti Sains Malaysia, Kelantan, Malaysia

^4^ Intelligent Systems Research Centre, School of Computing, Engineering and Intelligent Systems, Ulster University, Magee campus, Derry~Londonderry, BT48 7JL, UK.

^5^ Department of Radiology and Hotchkiss Brain Institute, University of Calgary, Calgary, AB, Canada.

^6^ The Bath Institute for the Augmented Human, University of Bath, Bath, BA2 7AY, UK.

^7^ Department of Neurosciences, School of Medical Sciences, Universiti Sains Malaysia, Kelantan, Malaysia

^8^ Centre for Intelligent Signal & Imaging Research (CISIR), Universiti Teknologi PETRONAS, Perak, Malaysia.

^©^Data used in preparation of this article were obtained from the Alzheimer’s Disease Neuroimaging Initiative (ADNI) database (adni.loni.usc.edu). As such, the investigators within the ADNI contributed to the design and implementation of ADNI and/or provided data but did not participate in analysis or writing of this report. A complete listing of ADNI investigators can be found at: <http://adni.loni.usc.edu/wpcontent/uploads/how_to_apply/ADNI_Acknowledgement_List.pdf>.


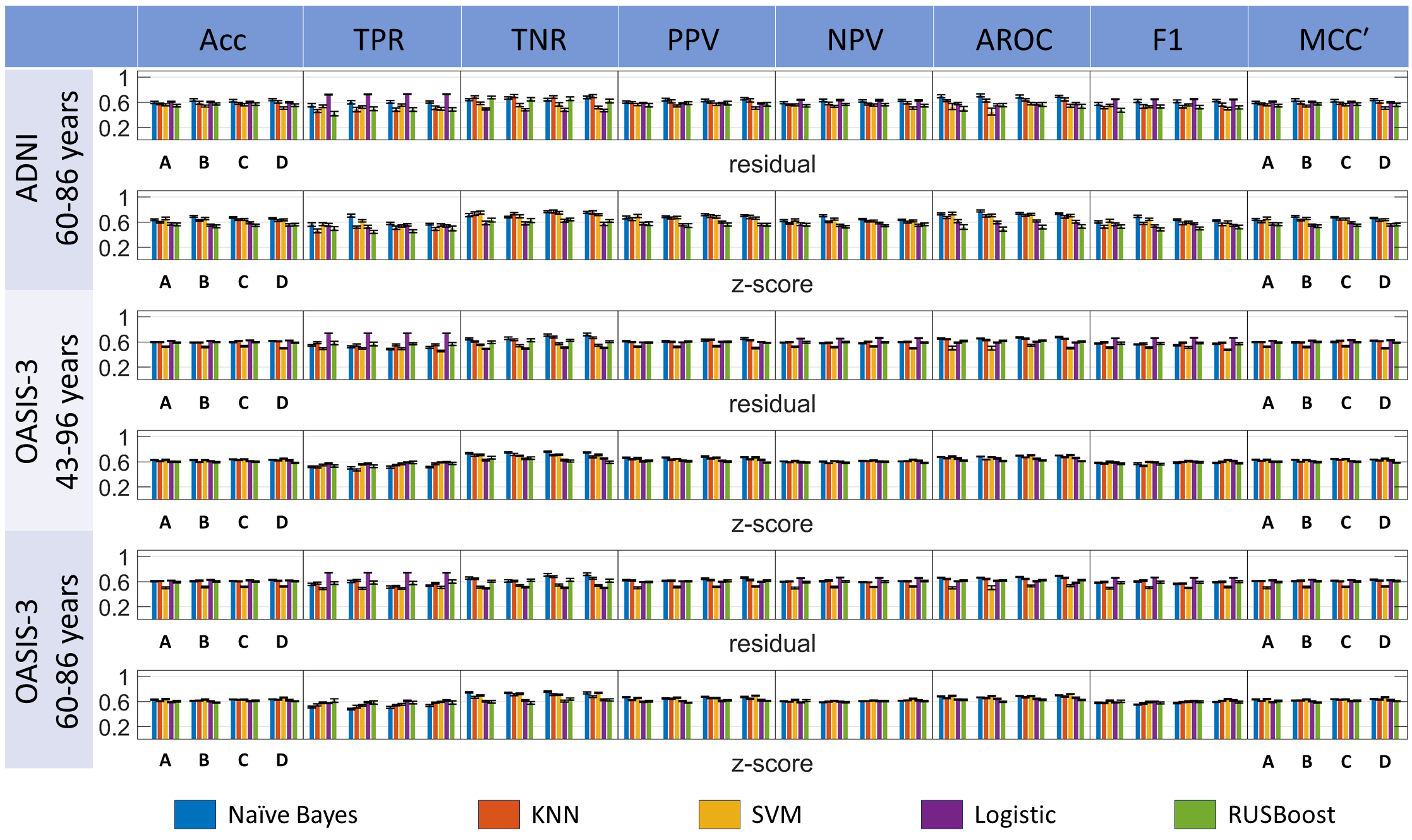


**Supplementary Figure 1:** Comparison among multiple classification pipeline options, involving five classifiers, four feature selection and two harmonization techniques. Performance is measured for randomly balanced cohorts extracted from ADNI and OASIS-3 imbalanced datasets within a Monte Carlo replication analysis. Results are presented for Naïve Bayes*,* KNN, SVM, Logistic and RUSBoost, residual and z-score harmonization procedures, as represented in the x-axis and legend labels. Bar groups denoted by letters **A-D** indicate the outcomes corresponding to the different features selected in the MCL analysis: (**A**) Features selected using the average scores (**Supplementary Table 13A** lists the feature labels); (**B**) Features selected based on the ReliefF criterion (**Supplementary Table 13B** lists the feature labels); (**C**) Features selected according to the combination of all evaluated feature selection algorithms (**Supplementary Table 13C** lists the feature labels); (**D**) Features selected within the SPSS statistical analysis (**Supplementary Table 13D** lists the feature labels). Column labels: **Acc**=accuracy. **TPR** = True Positive Rate. **TNR** = True Negative Rate. **PPV** = Positive Predictive Value. **NPV** = Negative Predictive Value. **AROC**=Area under Receiver Operating Curve. **F1**= F1 score, **MCC**=Matthew’s correlation coefficient. Performance metrics are normalized in the range [0, 1] and plotted together to enhance visual comparison.

**Supplementary Table 1:** Comparative analysis of classifier models and feature selection algorithms using different normalization techniques (see **Fig. 4** for complementary information) for the balanced analysis for ADNI dataset.

****classifier model pairwise comparison: p value post hoc test (Tukey’s HSD)

**** Pairwise comparison: p-value: Adjustment for multiple comparison: Bonferroni

**** NS = Not significant

***** Features selection methods:

A = Average score percentage (MCL app): chi-square, ReliefF, ANOVA & Kruskal Wallis

**B** = ReliefF (MCL app)

**C** = Frequent feature appearances from all feature ranking analysis

**D** = features extracted from statistical analysis (SPSS software): ANOVA, ANCOVA & Kruskal Wallis

| Dataset | Classification | Features selection group | Classifier model | Harmonization | Mean (%) | Standard deviation | Adjustment for multiple comparison: Bonferroni | | | Adjustment for multiple comparison: false discovery rate (FDR) Benjamini-Hochberg (𝛼 = 0.05) | | | |
| --- | --- | --- | --- | --- | --- | --- | --- | --- | --- | --- | --- | --- | --- |
|  |  |  |  |  |  |  | p-value (**Displaying only statistically significant p-values**) | 95% confidence interval | Pairwise comparison | Hypothesis ID | Raw p-value | adj. p-value | Pairwise comparison |
| ADNI | Accuracy | A | Naïve Bayes | Residual | 59.6875 | 7.7738 |  |  | NS for feature selection group |  |  |  | NS for feature selection group |
|  |  |  |  | z-score | 63.7500 | 5.9080 | < 0.001 | [0.0171, 0.0631] | Naïve Bayes > KNN | H4 | < 0.001 | 0.005 | Naïve Bayes > RUSBoost |
|  |  |  | KNN | Residual | 57.2917 | 5.7154 | < 0.001 | [0.0272, 0.0731] | Naïve Bayes > SVM | H3 | < 0.001 | 0.01 | Naïve Bayes > Logistic |
|  |  |  |  | z-score | 59.7917 | 6.1245 | < 0.001 | [0.0367, 0.0826] | Naïve Bayes > Logistic | H2 | < 0.001 | 0.015 | Naïve Bayes > SVM |
|  |  |  | SVM | Residual | 56.0417 | 7.4013 | < 0.001 | 0.0660, 0.1119] | Naïve Bayes > RUSBoost | H7 | < 0.001 | 0.02 | KNN > RUSBoost |
|  |  |  |  | z-score | 65.8333 | 8.8749 | < 0.001 | [0.0259, 0.0718] | KNN > RUSBoost | H1 | < 0.001 | 0.025 | Naïve Bayes > KNN |
|  |  |  | Logistic | Residual | 60.8333 | 3.4199 | < 0.001 | [0.0158, 0.0618] | SVM > RUSBoost | H9 | < 0.001 | 0.03 | SVM > RUSBoost |
|  |  |  |  | z-score | 57.1875 | 9.4744 | 0.005 | [0.0063, 0.0523] | Logistic > RUSBoost | H10 | < 0.001 | 0.035 | Logistic > RUSBoost |
|  |  |  | RUSBoost | Residual | 54.6875 | 9.4599 | < 0.001 | [-0.038, -0.017] | Residual < Z-score | H6 | 0.02 | 0.04 | KNN > Logistic |
|  |  |  |  | z-score | 56.4583 | 10.4035 |  |  |  | H1 | < 0.001 | 0.05 | Residual < Z-score |
|  |  | B | Naïve Bayes | Residual | 63.5417 | 9.2801 |  |  |  |  |  |  |  |
|  |  |  |  | z-score | 69.1667 | 6.5394 |  |  |  |  |  |  |  |
|  |  |  | KNN | Residual | 59.1667 | 9.9890 |  |  |  |  |  |  |  |
|  |  |  |  | z-score | 62.7083 | 5.6106 |  |  |  |  |  |  |  |
|  |  |  | SVM | Residual | 53.8542 | 6.4929 |  |  |  |  |  |  |  |
|  |  |  |  | z-score | 65.7292 | 7.6614 |  |  |  |  |  |  |  |
|  |  |  | Logistic | Residual | 60.4167 | 4.1115 |  |  |  |  |  |  |  |
|  |  |  |  | z-score | 55.2083 | 7.2010 |  |  |  |  |  |  |  |
|  |  |  | RUSBoost | Residual | 57.1875 | 7.8090 |  |  |  |  |  |  |  |
|  |  |  |  | z-score | 53.3333 | 9.8276 |  |  |  |  |  |  |  |
|  |  | C | Naïve Bayes | Residual | 62.3958 | 8.5090 |  |  |  |  |  |  |  |
|  |  |  |  | z-score | 67.3958 | 5.1599 |  |  |  |  |  |  |  |
|  |  |  | KNN | Residual | 58.2292 | 7.1365 |  |  |  |  |  |  |  |
|  |  |  |  | z-score | 63.9583 | 6.0494 |  |  |  |  |  |  |  |
|  |  |  | SVM | Residual | 56.0417 | 7.8506 |  |  |  |  |  |  |  |
|  |  |  |  | z-score | 64.2708 | 7.1621 |  |  |  |  |  |  |  |
|  |  |  | Logistic | Residual | 60.4167 | 4.7315 |  |  |  |  |  |  |  |
|  |  |  |  | z-score | 58.9583 | 6.2719 |  |  |  |  |  |  |  |
|  |  |  | RUSBoost | Residual | 56.9792 | 9.1506 |  |  |  |  |  |  |  |
|  |  |  |  | z-score | 55.0000 | 7.4474 |  |  |  |  |  |  |  |
|  |  | D | Naïve Bayes | Residual | 64.0625 | 7.4005 |  |  |  |  |  |  |  |
|  |  |  |  | z-score | 66.1458 | 4.3740 |  |  |  |  |  |  |  |
|  |  |  | KNN | Residual | 60.5208 | 8.9282 |  |  |  |  |  |  |  |
|  |  |  |  | z-score | 62.3958 | 7.1044 |  |  |  |  |  |  |  |
|  |  |  | SVM | Residual | 50.6250 | 8.0005 |  |  |  |  |  |  |  |
|  |  |  |  | z-score | 63.6458 | 6.7075 |  |  |  |  |  |  |  |
|  |  |  | Logistic | Residual | 59.8958 | 4.3214 |  |  |  |  |  |  |  |
|  |  |  |  | z-score | 55.5208 | 8.2306 |  |  |  |  |  |  |  |
|  |  |  | RUSBoost | Residual | 55.3125 | 10.0136 |  |  |  |  |  |  |  |
|  |  |  |  | z-score | 56.0417 | 8.6004 |  |  |  |  |  |  |  |
|  | F1 | A | Naïve Bayes | Residual | 57.4317 | 9.8654 |  |  | NS for feature selection group | H1 | 0.029 | 0.029 | A < B |
|  |  |  |  | z-score | 60.2462 | 8.0501 | < 0.001 | [0.0485, 0.1035] | Naïve Bayes > KNN | H4 | < 0.001 | 0.005 | Naïve Bayes > RUSBoost |
|  |  |  | KNN | Residual | 51.5039 | 7.6424 | < 0.001 | [0.0219, 0.0770] | Naïve Bayes > SVM | H10 | < 0.001 | 0.01 | Logistic > RUSBoost |
|  |  |  |  | z-score | 52.6057 | 9.8138 | < 0.001 | [0.0870, 0.1420] | Naïve Bayes > RUSBoost | H1 | < 0.001 | 0.015 | Naïve Bayes > KNN |
|  |  |  | SVM | Residual | 54.8608 | 8.1817 | < 0.001 | [-0.0809, -0.0259] | KNN < Logistic | H9 | < 0.001 | 0.02 | SVM > RUSBoost |
|  |  |  |  | z-score | 62.2155 | 10.1956 | 0.001 | [0.0110, 0.0660] | KNN > RUSBoost | H6 | < 0.001 | 0.025 | KNN < Logistic |
|  |  |  | Logistic | Residual | 64.9093 | 2.0702 | < 0.001 | [0.0375, 0.0926] | SVM > RUSBoost | H2 | < 0.001 | 0.03 | Naïve Bayes > SVM |
|  |  |  |  | z-score | 56.4569 | 9.3587 | < 0.001 | [0.0644, 0.1194] | Logistic > RUSBoost | H7 | < 0.001 | 0.035 | KNN > RUSBoost |
|  |  |  | RUSBoost | Residual | 47.1448 | 13.7933 |  |  | NS for harmonization | H8 | 0.008 | 0.04 | SVM < Logistic |
|  |  |  |  | z-score | 52.8764 | 12.5829 |  |  |  | H5 | 0.008 | 0.045 | KNN < SVM |
|  |  | B | Naïve Bayes | Residual | 62.0382 | 10.5443 |  |  |  | H3 | 0.025 | 0.05 | Naïve Bayes > Logistic |
|  |  |  |  | z-score | 69.2068 | 7.9010 |  |  |  |  |  |  | NS for harmonization |
|  |  |  | KNN | Residual | 53.3203 | 13.5570 |  |  |  |  |  |  |  |
|  |  |  |  | z-score | 57.9945 | 6.9649 |  |  |  |  |  |  |  |
|  |  |  | SVM | Residual | 52.9191 | 7.1089 |  |  |  |  |  |  |  |
|  |  |  |  | z-score | 64.3700 | 7.9005 |  |  |  |  |  |  |  |
|  |  |  | Logistic | Residual | 64.8133 | 2.5936 |  |  |  |  |  |  |  |
|  |  |  |  | z-score | 53.6583 | 8.2601 |  |  |  |  |  |  |  |
|  |  |  | RUSBoost | Residual | 53.1524 | 10.1856 |  |  |  |  |  |  |  |
|  |  |  |  | z-score | 48.4634 | 11.2111 |  |  |  |  |  |  |  |
|  |  | C | Naïve Bayes | Residual | 61.5740 | 9.4366 |  |  |  |  |  |  |  |
|  |  |  |  | z-score | 63.9038 | 6.2569 |  |  |  |  |  |  |  |
|  |  |  | KNN | Residual | 53.0155 | 9.8951 |  |  |  |  |  |  |  |
|  |  |  |  | z-score | 57.9984 | 9.5041 |  |  |  |  |  |  |  |
|  |  |  | SVM | Residual | 55.7485 | 7.2894 |  |  |  |  |  |  |  |
|  |  |  |  | z-score | 59.9889 | 8.1232 |  |  |  |  |  |  |  |
|  |  |  | Logistic | Residual | 64.9878 | 2.3530 |  |  |  |  |  |  |  |
|  |  |  |  | z-score | 57.4366 | 6.4739 |  |  |  |  |  |  |  |
|  |  |  | RUSBoost | Residual | 52.3661 | 12.1037 |  |  |  |  |  |  |  |
|  |  |  |  | z-score | 50.1003 | 9.2563 |  |  |  |  |  |  |  |
|  |  | D | Naïve Bayes | Residual | 62.6978 | 7.3766 |  |  |  |  |  |  |  |
|  |  |  |  | z-score | 62.5414 | 4.9028 |  |  |  |  |  |  |  |
|  |  |  | KNN | Residual | 56.0604 | 10.9740 |  |  |  |  |  |  |  |
|  |  |  |  | z-score | 56.3436 | 8.7631 |  |  |  |  |  |  |  |
|  |  |  | SVM | Residual | 49.9094 | 8.7740 |  |  |  |  |  |  |  |
|  |  |  |  | z-score | 60.0735 | 8.1566 |  |  |  |  |  |  |  |
|  |  |  | Logistic | Residual | 64.5919 | 2.6035 |  |  |  |  |  |  |  |
|  |  |  |  | z-score | 54.7249 | 7.8028 |  |  |  |  |  |  |  |
|  |  |  | RUSBoost | Residual | 51.7989 | 11.1857 |  |  |  |  |  |  |  |
|  |  |  |  | z-score | 52.1366 | 12.7316 |  |  |  |  |  |  |  |
|  | MCCʹ | A | Naïve Bayes | Residual | 59.7425 | 7.8510 |  |  | NS for feature selection group |  |  |  | NS for feature selection group |
|  |  |  |  | z-score | 64.3034 | 6.2119 | < 0.001 | [0.0142, 0.0610] | Naïve Bayes > KNN | H4 | < 0.001 | 0.005 | Naïve Bayes > RUSBoost |
|  |  |  | KNN | Residual | 57.5802 | 5.7863 | < 0.001 | [0.0273, 0.0742] | Naïve Bayes > SVM | H3 | < 0.001 | 0.01 | Naïve Bayes > Logistic |
|  |  |  |  | z-score | 60.4929 | 6.5552 | < 0.001 | [0.0366, 0.0835] | Naïve Bayes > Logistic | H7 | < 0.001 | 0.015 | KNN > RUSBoost |
|  |  |  | SVM | Residual | 56.0582 | 7.4115 | < 0.001 | [0.0663, 0.1132] | Naïve Bayes > RUSBoost | H2 | < 0.001 | 0.02 | Naïve Bayes > SVM |
|  |  |  |  | z-score | 66.2192 | 9.0403 | < 0.001 | [0.0287, 0.0756] | KNN > RUSBoost | H9 | < 0.001 | 0.025 | SVM > RUSBoost |
|  |  |  | Logistic | Residual | 61.1193 | 3.3306 | < 0.001 | [0.0155, 0.0624] | SVM > RUSBoost | H1 | < 0.001 | 0.03 | Naïve Bayes > KNN |
|  |  |  |  | z-score | 57.2190 | 9.4996 | 0.005 | [0.0062, 0.0531] | Logistic > RUSBoost | H10 | < 0.001 | 0.035 | Logistic > RUSBoost |
|  |  |  | RUSBoost | Residual | 54.7348 | 9.8026 | < 0.001 | [-0.040, -0.018] | Residual < Z-score | H6 | 0.009 | 0.04 | KNN > Logistic |
|  |  |  |  | z-score | 56.5636 | 10.5851 |  |  |  | H1 | < 0.001 | 0.05 | Residual < Z-score |
|  |  | B | Naïve Bayes | Residual | 63.5963 | 9.3091 |  |  |  |  |  |  |  |
|  |  |  |  | z-score | 69.2764 | 6.5585 |  |  |  |  |  |  |  |
|  |  |  | KNN | Residual | 59.4508 | 10.0564 |  |  |  |  |  |  |  |
|  |  |  |  | z-score | 63.1079 | 5.7277 |  |  |  |  |  |  |  |
|  |  |  | SVM | Residual | 53.8823 | 6.5564 |  |  |  |  |  |  |  |
|  |  |  |  | z-score | 65.8920 | 7.7067 |  |  |  |  |  |  |  |
|  |  |  | Logistic | Residual | 60.7032 | 4.1150 |  |  |  |  |  |  |  |
|  |  |  |  | z-score | 55.2654 | 7.2695 |  |  |  |  |  |  |  |
|  |  |  | RUSBoost | Residual | 57.3907 | 8.0443 |  |  |  |  |  |  |  |
|  |  |  |  | z-score | 53.4779 | 10.2273 |  |  |  |  |  |  |  |
|  |  | C | Naïve Bayes | Residual | 62.4446 | 8.5585 |  |  |  |  |  |  |  |
|  |  |  |  | z-score | 67.8335 | 5.3061 |  |  |  |  |  |  |  |
|  |  |  | KNN | Residual | 58.4681 | 7.2421 |  |  |  |  |  |  |  |
|  |  |  |  | z-score | 64.6844 | 6.3114 |  |  |  |  |  |  |  |
|  |  |  | SVM | Residual | 56.0591 | 7.9551 |  |  |  |  |  |  |  |
|  |  |  |  | z-score | 64.6948 | 7.3937 |  |  |  |  |  |  |  |
|  |  |  | Logistic | Residual | 60.7016 | 4.6615 |  |  |  |  |  |  |  |
|  |  |  |  | z-score | 59.0714 | 6.3156 |  |  |  |  |  |  |  |
|  |  |  | RUSBoost | Residual | 57.1610 | 9.6013 |  |  |  |  |  |  |  |
|  |  |  |  | z-score | 55.1329 | 7.7137 |  |  |  |  |  |  |  |
|  |  | D | Naïve Bayes | Residual | 64.1713 | 7.4711 |  |  |  |  |  |  |  |
|  |  |  |  | z-score | 66.5337 | 4.4947 |  |  |  |  |  |  |  |
|  |  |  | KNN | Residual | 60.8509 | 9.1927 |  |  |  |  |  |  |  |
|  |  |  |  | z-score | 63.1865 | 7.6431 |  |  |  |  |  |  |  |
|  |  |  | SVM | Residual | 50.6314 | 8.0519 |  |  |  |  |  |  |  |
|  |  |  |  | z-score | 63.8691 | 6.6921 |  |  |  |  |  |  |  |
|  |  |  | Logistic | Residual | 60.2011 | 4.2792 |  |  |  |  |  |  |  |
|  |  |  |  | z-score | 55.5883 | 8.2901 |  |  |  |  |  |  |  |
|  |  |  | RUSBoost | Residual | 55.5329 | 10.3346 |  |  |  |  |  |  |  |
|  |  |  |  | z-score | 56.1325 | 8.7837 |  |  |  |  |  |  |  |
|  | AROC | A | Naïve Bayes | Residual | 69.6042 | 10.7847 |  |  | NS for feature selection group |  |  |  | NS for feature selection group |
|  |  |  |  | z-score | 73.1458 | 6.3096 | < 0.001 | [0.0260, 0.0932] | Naïve Bayes > KNN | H4 | < 0.001 | 0.005 | Naïve Bayes > RUSBoost |
|  |  |  | KNN | Residual | 62.4792 | 6.3356 | < 0.001 | [0.0663, 13.3574] | Naïve Bayes > SVM | H7 | < 0.001 | 0.01 | KNN > RUSBoost |
|  |  |  |  | z-score | 67.3125 | 7.3963 | < 0.001 | [0.0978, 0.1650] | Naïve Bayes > Logistic | H3 | < 0.001 | 0.015 | Naïve Bayes > Logistic |
|  |  |  | SVM | Residual | 53.3333 | 20.6651 | < 0.001 | [0.1619, 0.2291] | Naïve Bayes > RUSBoost | H2 | < 0.001 | 0.02 | Naïve Bayes > SVM |
|  |  |  |  | z-score | 73.7083 | 8.5647 | 0.009 | [0.0067, 0.0740] | KNN > SVM | H9 | < 0.001 | 0.025 | SVM > RUSBoost |
|  |  |  | Logistic | Residual | 57.9167 | 8.0273 | < 0.001 | [0.0382, 0.1054] | KNN > Logistic | H6 | < 0.001 | 0.03 | KNN > Logistic |
|  |  |  |  | z-score | 61.6875 | 10.5846 | < 0.001 | [0.1023, 0.1695] | KNN > RUSBoost | H10 | < 0.001 | 0.035 | Logistic > RUSBoost |
|  |  |  | RUSBoost | Residual | 49.5625 | 15.2845 | < 0.001 | [0.0619, 0.1292] | SVM > RUSBoost | H1 | < 0.001 | 0.04 | Naïve Bayes > KNN |
|  |  |  |  | z-score | 52.2500 | 16.0029 | < 0.001 | [0.0305, 0.0977] | Logistic > RUSBoost | H5 | 0.001 | 0.045 | KNN > SVM |
|  |  | B | Naïve Bayes | Residual | 71.1042 | 10.7955 | < 0.001 | [-0.078, -0.047] | Residual < Z-score | H8 | 0.011 | 0.05 | SVM > Logistic |
|  |  |  |  | z-score | 77.7292 | 7.0756 |  |  |  | H1 | < 0.001 | 0.05 | Residual < Z-score |
|  |  |  | KNN | Residual | 62.6667 | 10.2637 |  |  |  |  |  |  |  |
|  |  |  |  | z-score | 70.0625 | 7.9060 |  |  |  |  |  |  |  |
|  |  |  | SVM | Residual | 45.0417 | 24.8349 |  |  |  |  |  |  |  |
|  |  |  |  | z-score | 70.6667 | 85.0095 |  |  |  |  |  |  |  |
|  |  |  | Logistic | Residual | 55.8333 | 7.9839 |  |  |  |  |  |  |  |
|  |  |  |  | z-score | 59.6250 | 9.6596 |  |  |  |  |  |  |  |
|  |  |  | RUSBoost | Residual | 55.5208 | 12.0579 |  |  |  |  |  |  |  |
|  |  |  |  | z-score | 48.8750 | 14.2932 |  |  |  |  |  |  |  |
|  |  | C | Naïve Bayes | Residual | 69.1042 | 11.2441 |  |  |  |  |  |  |  |
|  |  |  |  | z-score | 74.0833 | 6.3459 |  |  |  |  |  |  |  |
|  |  |  | KNN | Residual | 63.7708 | 7.8132 |  |  |  |  |  |  |  |
|  |  |  |  | z-score | 70.8542 | 6.9030 |  |  |  |  |  |  |  |
|  |  |  | SVM | Residual | 57.9167 | 8.8791 |  |  |  |  |  |  |  |
|  |  |  |  | z-score | 72.3333 | 6.5427 |  |  |  |  |  |  |  |
|  |  |  | Logistic | Residual | 57.4792 | 8.9971 |  |  |  |  |  |  |  |
|  |  |  |  | z-score | 61.7500 | 6.5834 |  |  |  |  |  |  |  |
|  |  |  | RUSBoost | Residual | 56.5417 | 13.6938 |  |  |  |  |  |  |  |
|  |  |  |  | z-score | 52.1250 | 13.3982 |  |  |  |  |  |  |  |
|  |  | D | Naïve Bayes | Residual | 69.5625 | 7.3790 |  |  |  |  |  |  |  |
|  |  |  |  | z-score | 73.5417 | 5.2904 |  |  |  |  |  |  |  |
|  |  |  | KNN | Residual | 64.6250 | 10.8560 |  |  |  |  |  |  |  |
|  |  |  |  | z-score | 68.4375 | 8.9261 |  |  |  |  |  |  |  |
|  |  |  | SVM | Residual | 54.4167 | 11.2556 |  |  |  |  |  |  |  |
|  |  |  |  | z-score | 70.5000 | 9.0620 |  |  |  |  |  |  |  |
|  |  |  | Logistic | Residual | 57.83333 | 8.3603 |  |  |  |  |  |  |  |
|  |  |  |  | z-score | 60.6458 | 10.3629 |  |  |  |  |  |  |  |
|  |  |  | RUSBoost | Residual | 53.4375 | 13.7316 |  |  |  |  |  |  |  |
|  |  |  |  | z-score | 53.1667 | 12.1954 |  |  |  |  |  |  |  |

**Supplementary Table 2:** Comparative analysis of classifier models and feature selection algorithms using different normalization techniques (see **Fig. 4** for complementary information) for a balanced analysis for OASIS-3 (age 60-86) dataset

| Dataset | Classification | Features selection group | Classifier model | Harmonization | Mean (%) | Standard deviation | Adjustment for multiple comparison: Bonferroni | | | Adjustment for multiple comparison: Benjamini-Hochberg | | | |
| --- | --- | --- | --- | --- | --- | --- | --- | --- | --- | --- | --- | --- | --- |
|  |  |  |  |  |  |  | p-value (**Displaying only statistically significant p-values**) | 95% confidence interval | Pairwise comparison | Hypothesis ID | Raw p-value | adj. p-value | Pairwise comparison |
| OASIS-3 (age 60-86) | Accuracy | A | Naïve Bayes | Residual | 60.7311 | 3.1284 | < 0.001 | [-0.02460, -0.0058] | A < D | H3 | < 0.001 | 0.0083 | A < D |
|  |  |  |  | z-score | 62.9953 | 3.2911 | < 0.001 | [-0.0236, -0.0048] | B < D | H5 | < 0.001 | 0.0167 | B < D |
|  |  |  | KNN | Residual | 61.0613 | 3.0008 | < 0.001 | [0.0310, 0.0533] | Naïve Bayes > SVM | H6 | 0.019 | 0.025 | C < D |
|  |  |  |  | z-score | 60.5660 | 2.3380 | < 0.001 | [0.0079, 0.0302] | Naïve Bayes > RUSBoost | H2 | < 0.001 | 0.005 | Naïve Bayes > SVM |
|  |  |  | SVM | Residual | 50.0943 | 5.9382 | < 0.001 | [0.0259, 0.0482] | KNN > SVM | H4 | < 0.001 | 0.01 | Naïve Baye s > RUSBoost |
|  |  |  |  | z-score | 63.8443 | 3.2402 | 0.006 | [0.0028, 0.0251] | KNN > RUSBoost | H5 | < 0.001 | 0.015 | KNN > SVM |
|  |  |  | Logistic | Residual | 61.8160 | 2.0842 | < 0.001 | [-0.0470, -0.0247] | SVM < Logistic | H7 | < 0.001 | 0.02 | KNN > RUSBoost |
|  |  |  |  | z-score | 59.1038 | 3.3322 | < 0.001 | [-0.0343, -0.0120] | SVM < RUSBoost | H8 | < 0.001 | 0.025 | SVM < Logistic |
|  |  |  | RUSBoost | Residual | 59.2217 | 4.4604 | 0.016 | [0.0016, 0.0239] | Logistic > RUSBoost | H9 | < 0.001 | 0.03 | SVM < RUSBoost |
|  |  |  |  | z-score | 60.5660 | 5.0210 | < 0.001 | [-0.031, -0.021] | Residual < Z-score | H10 | 0.002 | 0.035 | Logistic > RUSBoost |
|  |  | B | Naïve Bayes | Residual | 60.7075 | 3.4497 |  |  |  | H1 | < 0.001 | 0.05 | Residual < Z-score |
|  |  |  |  | z-score | 60.9906 | 2.5704 |  |  |  |  |  |  |  |
|  |  |  | KNN | Residual | 61.5802 | 4.3783 |  |  |  |  |  |  |  |
|  |  |  |  | z-score | 61.3915 | 2.7087 |  |  |  |  |  |  |  |
|  |  |  | SVM | Residual | 51.5802 | 5.4070 |  |  |  |  |  |  |  |
|  |  |  |  | z-score | 63.1368 | 3.2075 |  |  |  |  |  |  |  |
|  |  |  | Logistic | Residual | 62.8302 | 1.9333 |  |  |  |  |  |  |  |
|  |  |  |  | z-score | 60.2594 | 3.0641 |  |  |  |  |  |  |  |
|  |  |  | RUSBoost | Residual | 60.4009 | 4.4918 |  |  |  |  |  |  |  |
|  |  |  |  | z-score | 58.1604 | 3.6316 |  |  |  |  |  |  |  |
|  |  | C | Naïve Bayes | Residual | 61.1321 | 2.9013 |  |  |  |  |  |  |  |
|  |  |  |  | z-score | 63.2311 | 2.5948 |  |  |  |  |  |  |  |
|  |  |  | KNN | Residual | 60.4481 | 2.9129 |  |  |  |  |  |  |  |
|  |  |  |  | z-score | 62.5943 | 2.8347 |  |  |  |  |  |  |  |
|  |  |  | SVM | Residual | 51.7689 | 3.7138 |  |  |  |  |  |  |  |
|  |  |  |  | z-score | 63.0896 | 3.3917 |  |  |  |  |  |  |  |
|  |  |  | Logistic | Residual | 62.2642 | 2.4149 |  |  |  |  |  |  |  |
|  |  |  |  | z-score | 60.7783 | 4.8062 |  |  |  |  |  |  |  |
|  |  |  | RUSBoost | Residual | 60.2594 | 4.8198 |  |  |  |  |  |  |  |
|  |  |  |  | z-score | 61.0849 | 4.6833 |  |  |  |  |  |  |  |
|  |  | D | Naïve Bayes | Residual | 62.8066 | 3.1597 |  |  |  |  |  |  |  |
|  |  |  |  | z-score | 63.6085 | 2.2714 |  |  |  |  |  |  |  |
|  |  |  | KNN | Residual | 61.5094 | 3.8879 |  |  |  |  |  |  |  |
|  |  |  |  | z-score | 62.9717 | 2.7653 |  |  |  |  |  |  |  |
|  |  |  | SVM | Residual | 52.3585 | 4.1800 |  |  |  |  |  |  |  |
|  |  |  |  | z-score | 66.5802 | 2.9129 |  |  |  |  |  |  |  |
|  |  |  | Logistic | Residual | 62.0283 | 2.5859 |  |  |  |  |  |  |  |
|  |  |  |  | z-score | 62.0755 | 5.0974 |  |  |  |  |  |  |  |
|  |  |  | RUSBoost | Residual | 60.8019 | 4.5986 |  |  |  |  |  |  |  |
|  |  |  |  | z-score | 60.4717 | 3.1720 |  |  |  |  |  |  |  |
|  | F1 | A | Naïve Bayes | Residual | 58.5075 | 3.9593 | 0.020 | [-0.0259, -0.0015] | A < D | H6 | 0.001 | 0.0083 | C < D |
|  |  |  |  | z-score | 57.9142 | 5.1847 | 0.010 | [-.0270, -0.0026] | B < D | H5 | 0.002 | 0.0167 | B < D |
|  |  |  | KNN | Residual | 59.4643 | 4.4422 | 0.007 | [-0.0275, -0.0031] | C < D | H3 | 0.004 | 0.025 | A < D |
|  |  |  |  | z-score | 57.8484 | 4.0731 | < 0.001 | [0.0085, 0.0374] | Naïve Bayes > SVM | H2 | < 0.001 | 0.005 | Naïve Bayes > SVM |
|  |  |  | SVM | Residual | 49.5125 | 5.7867 | < 0.001 | [-0.0665, -0.0376] | Naïve Bayes < Logistic | H3 | < 0.001 | 0.01 | Naïve Bayes < Logistic |
|  |  |  |  | z-score | 61.5109 | 4.2382 | < 0.001 | [0.0191, 0.0480] | KNN > SVM | H5 | < 0.001 | 0.015 | KNN > SVM |
|  |  |  | Logistic | Residual | 66.0576 | 1.2930 | < 0.001 | [-0.0559, -0.0270] | KNN < Logistic | H6 | < 0.001 | 0.02 | KNN < Logistic |
|  |  |  |  | z-score | 58.4754 | 3.1268 | < 0.001 | [-0.0895, -0.0605] | SVM < Logistic | H8 | < 0.001 | 0.025 | SVM < Logistic |
|  |  |  | RUSBoost | Residual | 58.2931 | 6.2170 | < 0.001 | [-0.0484, -0.0195] | SVM < RUSBoost | H9 | < 0.001 | 0.03 | SVM < Logistic |
|  |  |  |  | z-score | 60.4379 | 7.3150 | < 0.001 | [0.0266, 0.0555] | Logistic > RUSBoost | H10 | < 0.001 | 0.035 | Logistic > RUSBoost |
|  |  | B | Naïve Bayes | Residual | 60.3393 | 4.2908 |  |  | NS for harmonization | H4 | 0.038 | 0.04 | Naïve Bayes < RUSBoost |
|  |  |  |  | z-score | 55.0534 | 3.7307 |  |  |  | H1 | 0.046 | 0.045 | Naïve Bayes < KNN |
|  |  |  | KNN | Residual | 61.6495 | 4.9947 |  |  |  |  |  |  | NS for harmonization |
|  |  |  |  | z-score | 56.9109 | 5.4564 |  |  |  |  |  |  |  |
|  |  |  | SVM | Residual | 50.4654 | 6.1437 |  |  |  |  |  |  |  |
|  |  |  |  | z-score | 59.1945 | 4.400 |  |  |  |  |  |  |  |
|  |  |  | Logistic | Residual | 66.6847 | 1.2132 |  |  |  |  |  |  |  |
|  |  |  |  | z-score | 59.7058 | 3.2938 |  |  |  |  |  |  |  |
|  |  |  | RUSBoost | Residual | 59.1552 | 7.0273 |  |  |  |  |  |  |  |
|  |  |  |  | z-score | 57.7608 | 6.7986 |  |  |  |  |  |  |  |
|  |  | C | Naïve Bayes | Residual | 56.6650 | 3.9187 |  |  |  |  |  |  |  |
|  |  |  |  | z-score | 57.6528 | 4.9979 |  |  |  |  |  |  |  |
|  |  |  | KNN | Residual | 57.1254 | 3.2750 |  |  |  |  |  |  |  |
|  |  |  |  | z-score | 59.0665 | 3.4155 |  |  |  |  |  |  |  |
|  |  |  | SVM | Residual | 50.2765 | 4.0691 |  |  |  |  |  |  |  |
|  |  |  |  | z-score | 59.8483 | 4.7931 |  |  |  |  |  |  |  |
|  |  |  | Logistic | Residual | 66.3434 | 1.5135 |  |  |  |  |  |  |  |
|  |  |  |  | z-score | 60.8942 | 4.5101 |  |  |  |  |  |  |  |
|  |  |  | RUSBoost | Residual | 58.8272 | 6.6679 |  |  |  |  |  |  |  |
|  |  |  |  | z-score | 59.7105 | 5.8883 |  |  |  |  |  |  |  |
|  |  | D | Naïve Bayes | Residual | 59.0026 | 2.9076 |  |  |  |  |  |  |  |
|  |  |  |  | z-score | 59.3751 | 3.4392 |  |  |  |  |  |  |  |
|  |  |  | KNN | Residual | 59.8176 | 4.0491 |  |  |  |  |  |  |  |
|  |  |  |  | z-score | 61.0869 | 2.6516 |  |  |  |  |  |  |  |
|  |  |  | SVM | Residual | 51.3963 | 5.6613 |  |  |  |  |  |  |  |
|  |  |  |  | z-score | 63.9394 | 3.4068 |  |  |  |  |  |  |  |
|  |  |  | Logistic | Residual | 66.1368 | 1.5895 |  |  |  |  |  |  |  |
|  |  |  |  | z-score | 61.8414 | 2.0448 |  |  |  |  |  |  |  |
|  |  |  | RUSBoost | Residual | 59.9989 | 6.9815 |  |  |  |  |  |  |  |
|  |  |  |  | z-score | 59.1212 | 6.3616 |  |  |  |  |  |  |  |
|  | MCCʹ | A | Naïve Bayes | Residual | 60.9037 | 3.2194 | < 0.001 | [-0.0251, -0.0061] | A < D | H3 | < 0.001 | 0.0083 | A < D |
|  |  |  |  | z-score | 63.3963 | 3.2959 | < 0.001 | [-0.0237, -0.0047] | B < D | H5 | < 0.001 | 0.0167 | B < D |
|  |  |  | KNN | Residual | 61.1478 | 2.9901 | < 0.001 | [0.0335, 0.0560] | Naïve Bayes > SVM | H6 | 0.025 | 0.025 | C < D |
|  |  |  |  | z-score | 60.7269 | 2.3645 | < 0.001 | [0.0096, 0.0321] | Naïve Bayes > RUSBoost | H2 | 0.048 | 0.033 | A < C |
|  |  |  | SVM | Residual | 50.0969 | 5.9472 | < 0.001 | [0.0264, 0.0490] | KNN > SVM | H2 | < 0.001 | 0.005 | Naïve Bayes > SVM |
|  |  |  |  | z-score | 63.9673 | 3.2327 | 0.008 | [0.0025, 0.0251] | KNN > RUSBoost | H4 | < 0.001 | 0.01 | Naïve Bayes > RUSBoost |
|  |  |  | Logistic | Residual | 62.1899 | 2.0293 | < 0.001 | [-0.0480, -0.0255] | SVM < Logistic | H5 | < 0.001 | 0.015 | KNN > SVM |
|  |  |  |  | z-score | 59.1253 | 3.3361 | < 0.001 | [-0.0352, -0.0126] | SVM < RUSBoost | H7 | < 0.001 | 0.02 | KNN > RUSBoost |
|  |  |  | RUSBoost | Residual | 59.2925 | 4.4720 | 0.016 | [0.0016, 0.0241] | Logistic > RUSBoost | H8 | < 0.001 | 0.025 | SVM < Logistic |
|  |  |  |  | z-score | 60.8073 | 5.2156 | < 0.001 | [-0.031, -0.021] | Residual < Z-score | H9 | < 0.001 | 0.03 | SVM < RUSBoost |
|  |  | B | Naïve Bayes | Residual | 60.8563 | 3.5670 |  |  |  | H10 | 0.002 | 0.035 | Logistic > RUSBoost |
|  |  |  |  | z-score | 61.4221 | 2.6536 |  |  |  | H1 | < 0.001 | 0.05 | Residual < Z-score |
|  |  |  | KNN | Residual | 61.6223 | 4.3815 |  |  |  |  |  |  |  |
|  |  |  |  | z-score | 61.7521 | 2.7153 |  |  |  |  |  |  |  |
|  |  |  | SVM | Residual | 51.5807 | 5.4335 |  |  |  |  |  |  |  |
|  |  |  |  | z-score | 63.4315 | 3.2211 |  |  |  |  |  |  |  |
|  |  |  | Logistic | Residual | 63.1784 | 1.8744 |  |  |  |  |  |  |  |
|  |  |  |  | z-score | 60.2994 | 3.0693 |  |  |  |  |  |  |  |
|  |  |  | RUSBoost | Residual | 60.5388 | 4.4808 |  |  |  |  |  |  |  |
|  |  |  |  | z-score | 58.3177 | 3.7034 |  |  |  |  |  |  |  |
|  |  | C | Naïve Bayes | Residual | 61.5758 | 3.1988 |  |  |  |  |  |  |  |
|  |  |  |  | z-score | 63.7251 | 2.4656 |  |  |  |  |  |  |  |
|  |  |  | KNN | Residual | 60.6363 | 3.0294 |  |  |  |  |  |  |  |
|  |  |  |  | z-score | 62.8547 | 2.9607 |  |  |  |  |  |  |  |
|  |  |  | SVM | Residual | 51.7812 | 3.7352 |  |  |  |  |  |  |  |
|  |  |  |  | z-score | 63.2867 | 3.3589 |  |  |  |  |  |  |  |
|  |  |  | Logistic | Residual | 62.6243 | 2.3485 |  |  |  |  |  |  |  |
|  |  |  |  | z-score | 60.7915 | 4.8096 |  |  |  |  |  |  |  |
|  |  |  | RUSBoost | Residual | 60.4857 | 4.9405 |  |  |  |  |  |  |  |
|  |  |  |  | z-score | 61.2148 | 4.7537 |  |  |  |  |  |  |  |
|  |  | D | Naïve Bayes | Residual | 63.1794 | 3.4229 |  |  |  |  |  |  |  |
|  |  |  |  | z-score | 64.0226 | 2.3940 |  |  |  |  |  |  |  |
|  |  |  | KNN | Residual | 61.6134 | 3.9225 |  |  |  |  |  |  |  |
|  |  |  |  | z-score | 61.6134 | 3.9225 |  |  |  |  |  |  |  |
|  |  |  | SVM | Residual | 52.3613 | 4.1964 |  |  |  |  |  |  |  |
|  |  |  |  | z-score | 66.7764 | 2.9571 |  |  |  |  |  |  |  |
|  |  |  | Logistic | Residual | 62.3784 | 2.5252 |  |  |  |  |  |  |  |
|  |  |  |  | z-score | 62.0895 | 5.1080 |  |  |  |  |  |  |  |
|  |  |  | RUSBoost | Residual | 61.0774 | 4.7127 |  |  |  |  |  |  |  |
|  |  |  |  | z-score | 60.6533 | 3.2087 |  |  |  |  |  |  |  |
|  | AROC | A | Naïve Bayes | Residual | 66.2378 | 2.1406 | 0.001 | [-0.0279, -0.0050] | A < D | H3 | < 0.001 | 0.0083 | A < D |
|  |  |  |  | z-score | 68.0236 | 3.1790 | < 0.001 | [-0.0293, -0.0063] | B < D | H5 | < 0.001 | 0.0167 | B < D |
|  |  |  | KNN | Residual | 64.1787 | 3.6558 | < 0.001 | [0.0097, 0.0369] | Naïve Bayes > KNN | H4 | 0.031 | 0.025 | B < C |
|  |  |  |  | z-score | 65.1333 | 2.9851 | < 0.001 | [0.0575, 0.0848] | Naïve Bayes > SVM | H1 | < 0.001 | 0.005 | Naïve Bayes > KNN |
|  |  |  | SVM | Residual | 50.1227 | 7.6377 | < 0.001 | [0.0410, 0.0683] | Naïve Bayes > Logistic | H2 | < 0.001 | 0.01 | Naïve Bayes > SVM |
|  |  |  |  | z-score | 69.0413 | 3.3664 | < 0.001 | [0.0445, 0.0718] | Naïve Bayes > RUSBoost | H3 | < 0.001 | 0.015 | Naïve Bayes > Logistic |
|  |  |  | Logistic | Residual | 60.6725 | 2.7434 | < 0.001 | [0.0342, 0.0615] | KNN > SVM | H4 | < 0.001 | 0.02 | Naïve Bayes > RUSBoost |
|  |  |  |  | z-score | 63.2366 | 3.7168 | < 0.001 | [0.0177, 0.0450] | KNN > Logistic | H5 | < 0.001 | 0.025 | KNN > SVM |
|  |  |  | RUSBoost | Residual | 61.7533 | 4.8717 | < 0.001 | [0.0212, 0.0485] | KNN > RUSBoost | H6 | < 0.001 | 0.03 | KNN > Logistic |
|  |  |  |  | z-score | 62.7525 | 4.4215 | 0.008 | [-0.0301, -0.0029] | SVM < Logistic | H7 | < 0.001 | 0.035 | KNN > RUSBoost |
|  |  | B | Naïve Bayes | Residual | 66.4306 | 3.1301 | < 0.001 | [-0.055, -0.043] | Residual < Z-score | H8 | < 0.001 | 0.04 | SVM < logistic |
|  |  |  |  | z-score | 66.6209 | 2.1095 |  |  |  | H9 | 0.009 | 0.045 | SVM < RUSBoost |
|  |  |  | KNN | Residual | 64.3955 | 4.5949 |  |  |  | H1 | < 0.001 | 0.005 | Residaul < Z-score |
|  |  |  |  | z-score | 65.4467 | 3.8292 |  |  |  |  |  |  |  |
|  |  |  | SVM | Residual | 50.5289 | 13.5394 |  |  |  |  |  |  |  |
|  |  |  |  | z-score | 68.8364 | 2.3262 |  |  |  |  |  |  |  |
|  |  |  | Logistic | Residual | 61.5450 | 3.9805 |  |  |  |  |  |  |  |
|  |  |  |  | z-score | 64.3124 | 3.2277 |  |  |  |  |  |  |  |
|  |  |  | RUSBoost | Residual | 61.9905 | 3.8556 |  |  |  |  |  |  |  |
|  |  |  |  | z-score | 59.6909 | 4.2791 |  |  |  |  |  |  |  |
|  |  | C | Naïve Bayes | Residual | 67.5523 | 3.1095 |  |  |  |  |  |  |  |
|  |  |  |  | z-score | 68.7035 | 2.3168 |  |  |  |  |  |  |  |
|  |  |  | KNN | Residual | 64.3360 | 3.1468 |  |  |  |  |  |  |  |
|  |  |  |  | z-score | 66.7634 | 3.4886 |  |  |  |  |  |  |  |
|  |  |  | SVM | Residual | 53.0719 | 5.9671 |  |  |  |  |  |  |  |
|  |  |  |  | z-score | 68.7645 | 3.8384 |  |  |  |  |  |  |  |
|  |  |  | Logistic | Residual | 60.6240 | 3.7212 |  |  |  |  |  |  |  |
|  |  |  |  | z-score | 64.4081 | 4.4113 |  |  |  |  |  |  |  |
|  |  |  | RUSBoost | Residual | 62.3486 | 4.2655 |  |  |  |  |  |  |  |
|  |  |  |  | z-score | 62.8287 | 5.1086 |  |  |  |  |  |  |  |
|  |  | D | Naïve Bayes | Residual | 69.2295 | 3.1924 |  |  |  |  |  |  |  |
|  |  |  |  | z-score | 69.9446 | 2.3887 |  |  |  |  |  |  |  |
|  |  |  | KNN | Residual | 65.9151 | 4.0882 |  |  |  |  |  |  |  |
|  |  |  |  | z-score | 67.9481 | 3.4249 |  |  |  |  |  |  |  |
|  |  |  | SVM | Residual | 53.4438 | 6.4185 |  |  |  |  |  |  |  |
|  |  |  |  | z-score | 72.0124 | 2.3998 |  |  |  |  |  |  |  |
|  |  |  | Logistic | Residual | 58.1742 | 3.4906 |  |  |  |  |  |  |  |
|  |  |  |  | z-score | 66.0674 | 5.1594 |  |  |  |  |  |  |  |
|  |  |  | RUSBoost | Residual | 62.4612 | 4.1880 |  |  |  |  |  |  |  |
|  |  |  |  | z-score | 62.4023 | 3.5042 |  |  |  |  |  |  |  |


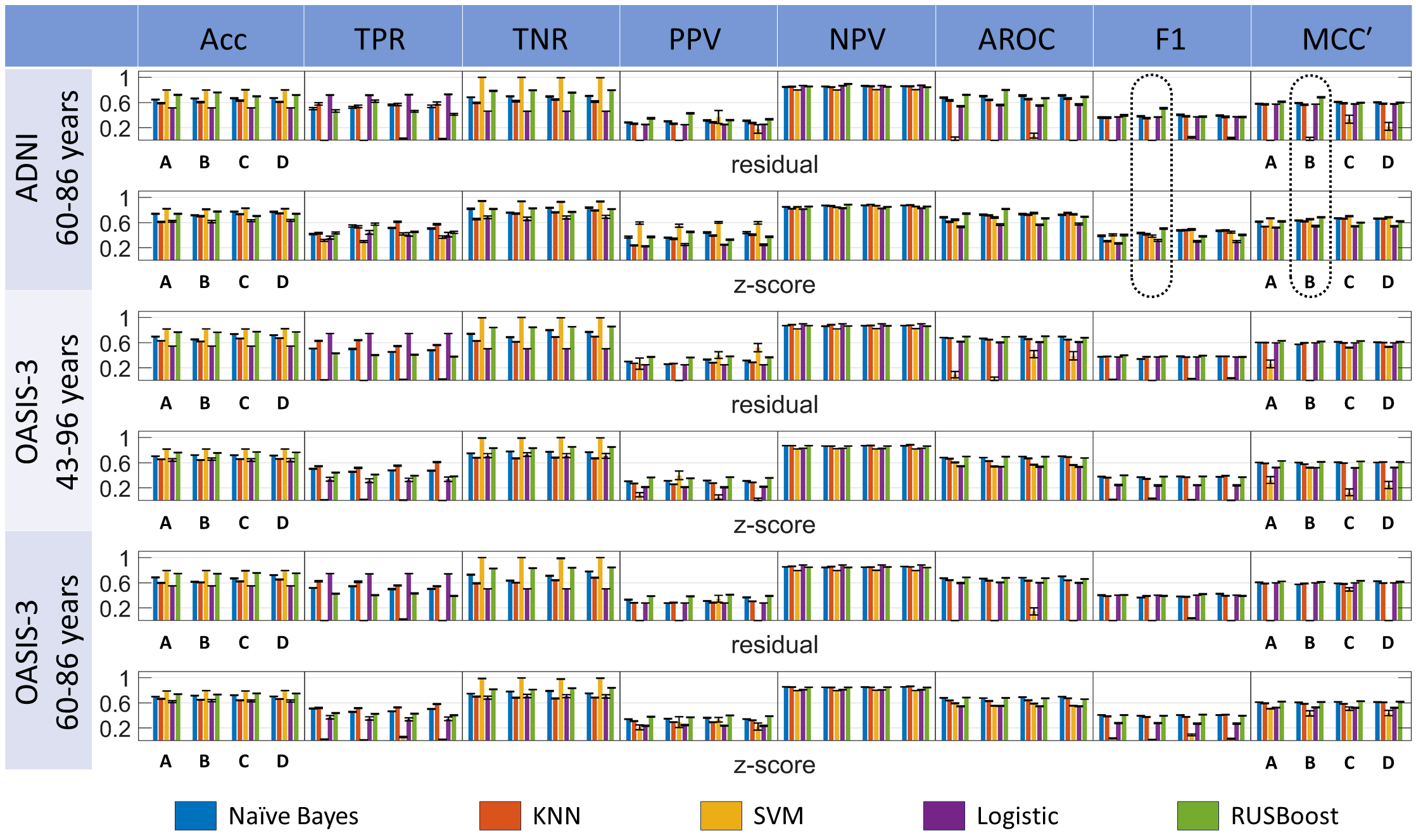


**Supplementary Figure 2:** Comparison among multiple classification pipeline options, involving five classifiers, four feature selection, and two harmonization techniques. Performance is measured directly for ADNI and OASIS-3 imbalanced datasets within a Monte Carlo replication analysis. See **Supplementary Figure 1** for complementary information regarding balanced data analysis and figure caption.

**Supplementary Table 3:** Comparative analysis of classifier models and feature selection algorithms using different normalization techniques (see **Fig. 5** for complementary information) for imbalanced analysis for ADNI dataset

****classifier model pairwise comparison: p value post hoc test (Tukey’s HSD)

**** Pairwise comparison: p-value: Adjustment for multiple comparison: Bonferroni

**** NS = Not significant

***** Features selection methods:

A = Average score percentage (MCL app): chi-square, ReliefF, ANOVA & Kruskal Wallis

**B** = ReliefF (MCL app)

**C** = Frequent feature appearances from all feature ranking analysis

**D** = features extracted from statistical analysis (SPSS software): ANOVA, ANCOVA & Kruskal Wallis

| Dataset | Classification | Features selection group | Classifier model | Harmonization | Mean (%) | Standard deviation | Adjustment for multiple comparison: Bonferroni | | | Adjustment for multiple comparison: Benjamini-Hochberg | | | |
| --- | --- | --- | --- | --- | --- | --- | --- | --- | --- | --- | --- | --- | --- |
|  |  |  |  |  |  |  | p-value (**Displaying only statistically significant p-values**) | 95% confidence interval | Pairwise comparison | Hypothesis ID | Raw p-value | adj. p-value | Pairwise comparison |
| ADNI | Accuracy | A | Naïve Bayes | Residual | 64.7521 | 2.3109 | < 0.001 | [-0.0243, -0.0078] | A < B | H3 | < 0.001 | 0.0083 | A < D |
|  |  |  |  | z-score | 73.9256 | 3.3967 | < 0.001 | [-0.0265, -0.0099] | A < C | H2 | <0.001 | 0.0167 | A < C |
|  |  |  | KNN | Residual | 59.0909 | 3.7230 | < 0.001 | [-0.0312, -0.0146] | A < D | H1 | < 0.001 | 0.025 | A < B |
|  |  |  |  | z-score | 61.0744 | 3.8754 | < 0.001 | [0.0430, 0.0626] | Naïve Bayes > KNN | H5 | 0.033 | 0.0333 | B < D |
|  |  |  | SVM | Residual | 80.1653 | 0 | < 0.001 | [-0.1143, -0.0946] | Naïve Bayes < SVM | H1 | < 0.001 | 0.005 | Naïve Bayes > KNN |
|  |  |  |  | z-score | 81.9008 | 1.2831 | < 0.001 | [0.1272, 0.1469] | Naïve Bayes > Logistic | H2 | < 0.001 | 0.01 | Naïve Bayes < SVM |
|  |  |  | Logistic | Residual | 51.4463 | 0.4546 | < 0.001 | [-0.0364, -0.0167] | Naïve Bayes < RUSBoost | H3 | < 0.001 | 0.015 | Naïve Bayes > Logistic |
|  |  |  |  | z-score | 62.0248 | 5.9685 | < 0.001 | [-0.1671, -0.1474] | KNN < SVM | H4 | < 0.001 | 0.02 | Naïve Bayes < -RUSBoost |
|  |  |  | RUSBoost | Residual | 72.1488 | 2.5733 | < 0.001 | [0.0744, 0.0941] | KNN > Logistic | H5 | < 0.001 | 0.025 | KNN < SVM |
|  |  |  |  | z-score | 74.0909 | 2.6446 | < 0.001 | [-0.0892, -0.0695] | KNN < RUSBoost | H6 | < 0.001 | 0.03 | KNN > Logistic |
|  |  | B | Naïve Bayes | Residual | 66.4050 | 2.2317 | < 0.001 | [0.2317, 0.2513] | SVM > Logistic | H7 | < 0.001 | 0.035 | KNN < RUSBoost |
|  |  |  |  | z-score | 71.6942 | 2.4703 | < 0.001 | [0.0681, 0.0877] | SVM > RUSBoost | H9 | < 0.001 | 0.04 | SVM > Logistic |
|  |  |  | KNN | Residual | 60.5372 | 3.8566 | < 0.001 | [-0.1734, -0.1538] | Logistic < RUSBoost | H9 | < 0.001 | 0.045 | SVM > RUSBoost |
|  |  |  |  | z-score | 70.1240 | 3.8041 | < 0.001 | [-0.069, -0.06] | Residual < Z-score | H10 | < 0.001 | 0.05 | Logistic < RUSBoost |
|  |  |  | SVM | Residual | 80.0826 | 0.3696 |  |  |  | H1 | < 0.001 | 0.05 | Residual < Z-score |
|  |  |  |  | z-score | 81.1157 | 2.2478 |  |  |  |  |  |  |  |
|  |  |  | Logistic | Residual | 51.3223 | 0.3696 |  |  |  |  |  |  |  |
|  |  |  |  | z-score | 61.6942 | 8.0720 |  |  |  |  |  |  |  |
|  |  |  | RUSBoost | Residual | 76.0331 | 2.3681 |  |  |  |  |  |  |  |
|  |  |  |  | z-score | 77.6446 | 2.3448 |  |  |  |  |  |  |  |
|  |  | C | Naïve Bayes | Residual | 66.9421 | 3.1384 |  |  |  |  |  |  |  |
|  |  |  |  | z-score | 77.3554 | 2.3742 |  |  |  |  |  |  |  |
|  |  |  | KNN | Residual | 63.0992 | 3.6987 |  |  |  |  |  |  |  |
|  |  |  |  | z-score | 73.4298 | 3.0487 |  |  |  |  |  |  |  |
|  |  |  | SVM | Residual | 80.2893 | 0.9003 |  |  |  |  |  |  |  |
|  |  |  |  | z-score | 82.9339 | 1.5925 |  |  |  |  |  |  |  |
|  |  |  | Logistic | Residual | 51.4050 | 0.6889 |  |  |  |  |  |  |  |
|  |  |  |  | z-score | 62.8512 | 6.7059 |  |  |  |  |  |  |  |
|  |  |  | RUSBoost | Residual | 69.8347 | 2.2988 |  |  |  |  |  |  |  |
|  |  |  |  | z-score | 70.6612 | 2.9434 |  |  |  |  |  |  |  |
|  |  | D | Naïve Bayes | Residual | 67.1488 | 1.7962 |  |  |  |  |  |  |  |
|  |  |  |  | z-score | 77.1074 | 3.4661 |  |  |  |  |  |  |  |
|  |  |  | KNN | Residual | 60.9091 | 3.6970 |  |  |  |  |  |  |  |
|  |  |  |  | z-score | 74.8347 | 2.8938 |  |  |  |  |  |  |  |
|  |  |  | SVM | Residual | 80.1653 | 0.5363 |  |  |  |  |  |  |  |
|  |  |  |  | z-score | 82.2314 | 1.8087 |  |  |  |  |  |  |  |
|  |  |  | Logistic | Residual | 51.5289 | 0.4852 |  |  |  |  |  |  |  |
|  |  |  |  | z-score | 63.4298 | 6.5178 |  |  |  |  |  |  |  |
|  |  |  | RUSBoost | Residual | 72.0248 | 1.8822 |  |  |  |  |  |  |  |
|  |  |  |  | z-score | 74.1322 | 2.3847 |  |  |  |  |  |  |  |
|  | F1 | A | Naïve Bayes | Residual | 36.0377 | 4.4481 | < 0.001 | [-0.0522, -0.0281] | A < B | H2 | < 0.001 | 0.0083 | A < C |
|  |  |  |  | z-score | 38.9049 | 4.7583 | < 0.001 | [-0.0568, -0.0328] | A < C | H1 | < 0.001 | 0.0167 | A < B |
|  |  |  | KNN | Residual | 35.9312 | 4.8959 | < 0.001 | [-0.0501, -0.0261] | A < D | H3 | < 0.001 | 0.025 | A < D |
|  |  |  |  | z-score | 30.4679 | 3.5664 | < 0.001 | [0.0068, 0.0354] | Naïve Bayes > KNN | H9 | < 0.001 | 0.005 | SVM < Logistic |
|  |  |  | SVM | Residual | 0 | 0 | < 0.001 | [0.1728, 0.2014] | Naïve Bayes > SVM | H2 | < 0.001 | 0.01 | Naïve Bayes > SVM |
|  |  |  |  | z-score | 40.5470 | 6.0897 | < 0.001 | [0.0650, 0.0936] | Naïve Bayes > Logistic | H5 | < 0.001 | 0.015 | KNN > SVM |
|  |  |  | Logistic | Residual | 37.0613 | 0.8350 | < 0.001 | [0.1517, 0.1803] | KNN > SVM | H8 | < 0.001 | 0.02 | SVM < Logistic |
|  |  |  |  | z-score | 26.9913 | 4.6160 | < 0.001 | [0.0440, 0.0725] | KNN > Logistic | H10 | < 0.001 | 0.025 | Logistic < RUSBoost |
|  |  |  | RUSBoost | Residual | 39.6844 | 5.3813 | < 0.001 | [-0.0392, -0.0106] | KNN < RUSBoost | H3 | < 0.001 | 0.03 | Logistic > Logistic |
|  |  |  |  | z-score | 39.9543 | 5.7453 | < 0.001 | [-0.1221, -0.0935] | SVM < Logistic | H6 | < 0.001 | 0.035 | KNN > Logistic |
|  |  | B | Naïve Bayes | Residual | 38.1444 | 3.3950 | < 0.001 | [-0.2052, -0.1766] | SVM < RUSBoost | H7 | < 0.001 | 0.04 | KNN < RUSBoost |
|  |  |  |  | z-score | 43.2623 | 4.6927 | < 0.001 | [-0.0975, 0.0689] | Logistic < RUSBoost | H1 | < 0.001 | 0.045 | Naïve Bayes > KNN |
|  |  |  | KNN | Residual | 35.1090 | 4.7882 | < 0.001 | [-0.098, -0.085] | Residual < Z-score | H1 | < 0.001 | 0.05 | Residual < Z-score |
|  |  |  |  | z-score | 41.4994 | 5.0098 |  |  |  |  |  |  |  |
|  |  |  | SVM | Residual | 0 | 0 |  |  |  |  |  |  |  |
|  |  |  |  | z-score | 38.4188 | 6.8875 |  |  |  |  |  |  |  |
|  |  |  | Logistic | Residual | 36.8673 | 0.6923 |  |  |  |  |  |  |  |
|  |  |  |  | z-score | 31.3866 | 6.9799 |  |  |  |  |  |  |  |
|  |  |  | RUSBoost | Residual | 50.5969 | 5.2048 |  |  |  |  |  |  |  |
|  |  |  |  | z-score | 50.4421 | 5.0576 |  |  |  |  |  |  |  |
|  |  | C | Naïve Bayes | Residual | 40.3730 | 3.9487 |  |  |  |  |  |  |  |
|  |  |  |  | z-score | 47.4765 | 3.9164 |  |  |  |  |  |  |  |
|  |  |  | KNN | Residual | 37.9349 | 4.4667 |  |  |  |  |  |  |  |
|  |  |  |  | z-score | 47.9604 | 3.6313 |  |  |  |  |  |  |  |
|  |  |  | SVM | Residual | 4.6210 | 5.7673 |  |  |  |  |  |  |  |
|  |  |  |  | z-score | 49.0687 | 5.9858 |  |  |  |  |  |  |  |
|  |  |  | Logistic | Residual | 37.1773 | 0.9580 |  |  |  |  |  |  |  |
|  |  |  |  | z-score | 30.1444 | 4.7325 |  |  |  |  |  |  |  |
|  |  |  | RUSBoost | Residual | 37.5764 | 3.9190 |  |  |  |  |  |  |  |
|  |  |  |  | z-score | 38.0492 | 3.5583 |  |  |  |  |  |  |  |
|  |  | D | Naïve Bayes | Residual | 39.2905 | 4.3668 |  |  |  |  |  |  |  |
|  |  |  |  | z-score | 46.9732 | 4.8702 |  |  |  |  |  |  |  |
|  |  |  | KNN | Residual | 37.1536 | 4.7976 |  |  |  |  |  |  |  |
|  |  |  |  | z-score | 47.5438 | 4.3277 |  |  |  |  |  |  |  |
|  |  |  | SVM | Residual | 3.3535 | 5.6149 |  |  |  |  |  |  |  |
|  |  |  |  | z-score | 44.7706 | 6.3947 |  |  |  |  |  |  |  |
|  |  |  | Logistic | Residual | 37.3687 | 0.8824 |  |  |  |  |  |  |  |
|  |  |  |  | z-score | 30.0029 | 6.9896 |  |  |  |  |  |  |  |
|  |  |  | RUSBoost | Residual | 36.8080 | 4.9263 |  |  |  |  |  |  |  |
|  |  |  |  | z-score | 40.4196 | 5.3271 |  |  |  |  |  |  |  |
|  | MCCʹ | A | Naïve Bayes | Residual | 57.7349 | 30945 | < 0.001 | [-0.0465, -0.0097] | A < B | H2 | < 0.001 | 0.0083 | A < C |
|  |  |  |  | z-score | 61.3445 | 3.3174 | < 0.001 | [-0.0769, -0.0402] | A < C | H3 | < 0.001 | 0.0167 | A < D |
|  |  |  | KNN | Residual | 56.9601 | 3.9306 | < 0.001 | [-0.0644, -0.0277] | A < D | H4 | < 0.001 | 0.025 | B < C |
|  |  |  |  | z-score | 53.5664 | 2.7129 | < 0.001 | [-0.0488, -0.0121] | B < C | H1 | < 0.001 | 0.033 | A < B |
|  |  |  | SVM | Residual | 0 | 0 | < 0.001 | [0.1874, 0.2310] | Naïve Bayes > SVM | H5 | 0.012 | 0.042 | B < D |
|  |  |  |  | z-score | 66.7977 | 2.5259 | < 0.001 | [0.0420, 0.0856] | Naïve Bayes > Logistic | H9 | < 0.001 | 0.005 | SVM < RUSBoost |
|  |  |  | Logistic | Residual | 57.4274 | 0.8174 | < 0.001 | [0.1660, 0.2096] | KNN > SVM | H2 | < 0.001 | 0.01 | Naïve Bayes > SVM |
|  |  |  |  | z-score | 51.9918 | 3.0235 | < 0.001 | [0.0205, 0.0641] | KNN > Logistic | H5 | < 0.001 | 0.015 | KNN > SVM |
|  |  |  | RUSBoost | Residual | 61.2951 | 3.4053 | 0.004 | [-0.0501, -0.0065] | KNN < RUSBoost | H8 | < 0.001 | 0.02 | SVM < Logistic |
|  |  |  |  | z-score | 61.8886 | 3.6280 | < 0.001 | [-0.1672, -0.1236] | SVM < Logistic | H10 | < 0.001 | 0.025 | Logistic < RUSBoost |
|  |  | B | Naïve Bayes | Residual | 59.3151 | 2.3941 | < 0.001 | [-0.2379, -0.1943] | SVM < RUSBoost | H3 | < 0.001 | 0.03 | Naïve Bayes > Logistic |
|  |  |  |  | z-score | 63.2723 | 3.0799 | < 0.001 | [-0.0925, -0.0489] | Logistic < RUSBoost | H6 | < 0.001 | 0.035 | KNN > Logistic |
|  |  |  | KNN | Residual | 56.5625 | 3.5708 | < 0.001 | [-0.129, -0.109] | Residual < Z-score | H7 | < 0.001 | 0.04 | KNN < RUSBoost |
|  |  |  |  | z-score | 61.9587 | 3.5055 |  |  |  | H1 | 0.007 | 0.045 | Naïve Bayes > KNN |
|  |  |  | SVM | Residual | 2.3388 | 10.4594 |  |  |  | H1 | < 0.001 | 0.05 | Residual < Z-score |
|  |  |  |  | z-score | 65.2231 | 4.1998 |  |  |  |  |  |  |  |
|  |  |  | Logistic | Residual | 57.2378 | 0.6779 |  |  |  |  |  |  |  |
|  |  |  |  | z-score | 54.4625 | 5.0155 |  |  |  |  |  |  |  |
|  |  |  | RUSBoost | Residual | 68.3117 | 3.4468 |  |  |  |  |  |  |  |
|  |  |  |  | z-score | 68.4252 | 3.2375 |  |  |  |  |  |  |  |
|  |  | C | Naïve Bayes | Residual | 60.7965 | 2.9720 |  |  |  |  |  |  |  |
|  |  |  |  | z-score | 66.6945 | 2.6367 |  |  |  |  |  |  |  |
|  |  |  | KNN | Residual | 58.8082 | 3.3697 |  |  |  |  |  |  |  |
|  |  |  |  | z-score | 66.3830 | 2.5412 |  |  |  |  |  |  |  |
|  |  |  | SVM | Residual | 33.5233 | 28.4519 |  |  |  |  |  |  |  |
|  |  |  |  | z-score | 70.2082 | 3.1579 |  |  |  |  |  |  |  |
|  |  |  | Logistic | Residual | 57.5376 | 0.9422 |  |  |  |  |  |  |  |
|  |  |  |  | z-score | 54.1100 | 3.0002 |  |  |  |  |  |  |  |
|  |  |  | RUSBoost | Residual | 59.5593 | 2.6148 |  |  |  |  |  |  |  |
|  |  |  |  | z-score | 59.9720 | 02.6111 |  |  |  |  |  |  |  |
|  |  | D | Naïve Bayes | Residual | 60.2132 | 2.9667 |  |  |  |  |  |  |  |
|  |  |  |  | z-score | 66.3907 | 3.4642 |  |  |  |  |  |  |  |
|  |  |  | KNN | Residual | 58.1145 | 3.5570 |  |  |  |  |  |  |  |
|  |  |  |  | z-score | 66.2494 | 2.9748 |  |  |  |  |  |  |  |
|  |  |  | SVM | Residual | 21.8907 | 27.6870 |  |  |  |  |  |  |  |
|  |  |  |  | z-score | 68.4077 | 3.1179 |  |  |  |  |  |  |  |
|  |  |  | Logistic | Residual | 57.7281 | 0.8641 |  |  |  |  |  |  |  |
|  |  |  |  | z-score | 54.2344 | 4.0943 |  |  |  |  |  |  |  |
|  |  |  | RUSBoost | Residual | 59.662 | 3.0223 |  |  |  |  |  |  |  |
|  |  |  |  | z-score | 62.1512 | 3.3414 |  |  |  |  |  |  |  |
|  | AROC | A | Naïve Bayes | Residual | 67.7181 | 3.4287 | < 0.001 | [-0.0529, -0.0259] | A < B | H1 | < 0.001 | 0.0083 | A < B |
|  |  |  |  | z-score | 68.4412 | 3.5404 | < 0.001 | [-0.0421, -0.0152] | A < C | H3 | < 0.001 | 0.0167 | A < D |
|  |  |  | KNN | Residual | 63.4806 | 5.0820 | < 0.001 | [-0.0437, -0.0168] | A < D | H2 | < 0.001 | 0.025 | A < C |
|  |  |  |  | z-score | 61.0856 | 4.6171 | < 0.001 | [0.0174, 0.0494] | Naïve Bayes > KNN | H4 | 0.041 | 0.033 | B > C |
|  |  |  | SVM | Residual | 2.5 | 11.1803 | < 0.001 | [0.3292, 0.3612] | Naïve Bayes > SVM | H9 | < 0.001 | 0.005 | SVM < RUSBoost |
|  |  |  |  | z-score | 64.4907 | 5.6290 | < 0.001 | [0.1351, 0.1671] | Naïve Bayes > Logistic | H2 | < 0.001 | 0.01 | Naïve Bayes > SVM |
|  |  |  | Logistic | Residual | 53.9708 | 3.9789 | < 0.001 | [0.2958, 0.3278] | KNN > SVM | H5 | < 0.001 | 0.015 | KNN > SVM |
|  |  |  |  | z-score | 53.0991 | 5.3586 | < 0.001 | [0.1016, 0.1337] | KNN > Logistic | H8 | < 0.001 | 0.02 | SVM < Logistic |
|  |  |  | RUSBoost | Residual | 72.1981 | 3.5397 | < 0.001 | [-0.0662, -0.0342] | KNN < RUSBoost | H10 | < 0.001 | 0.025 | Logistic < RUSBoost |
|  |  |  |  | z-score | 74.3435 | 4.1253 | < 0.001 | [-0.2101, -0.1781] | SVM < Logistic | H3 | < 0.001 | 0.03 | Naïve Bayes > Logistic |
|  |  | B | Naïve Bayes | Residual | 69.9653 | 3.7476 | < 0.001 | [-0.3780, -0.3460] | SVM < RUSBoost | H6 | < 0.001 | 0.035 | KNN > Logistic |
|  |  |  |  | z-score | 72.3981 | 4.3473 | < 0.001 | [-0.1839, -0.1518] | Logistic < RUSBoost | H7 | < 0.001 | 0.04 | KNN < RUSBoost |
|  |  |  | KNN | Residual | 64.0995 | 4.1817 | < 0.001 | [0.524, 0.535] | Residual < Z-score | H1 | < 0.001 | 0.045 | Naïve Bayes > KNN |
|  |  |  |  | z-score | 71.5222 | 3.8474 |  |  |  | H4 | 0.004 | 0.05 | Naïve Bayes < RUSBoost |
|  |  |  | SVM | Residual | 0 | 0 |  |  |  | H1 | < 0.001 | 0.05 | Residual < Z-score |
|  |  |  |  | z-score | 68.0750 | 4.7735 |  |  |  |  |  |  |  |
|  |  |  | Logistic | Residual | 56.1176 | 4.3962 |  |  |  |  |  |  |  |
|  |  |  |  | z-score | 56.8838 | 5.7037 |  |  |  |  |  |  |  |
|  |  |  | RUSBoost | Residual | 80.1088 | 2.5231 |  |  |  |  |  |  |  |
|  |  |  |  | z-score | 81.5407 | 2.9199 |  |  |  |  |  |  |  |
|  |  | C | Naïve Bayes | Residual | 71.0606 | 4.6236 |  |  |  |  |  |  |  |
|  |  |  |  | z-score | 73.4620 | 4.3962 |  |  |  |  |  |  |  |
|  |  |  | KNN | Residual | 65.2917 | 4.0369 |  |  |  |  |  |  |  |
|  |  |  |  | z-score | 72.7023 | 3.7043 |  |  |  |  |  |  |  |
|  |  |  | SVM | Residual | 7.3 | 17.8447 |  |  |  |  |  |  |  |
|  |  |  |  | z-score | 75.0125 | 5.0479 |  |  |  |  |  |  |  |
|  |  |  | Logistic | Residual | 55.0972 | 4.4881 |  |  |  |  |  |  |  |
|  |  |  |  | z-score | 56.4222 | 4.3605 |  |  |  |  |  |  |  |
|  |  |  | RUSBoost | Residual | 66.9606 | 3.5426 |  |  |  |  |  |  |  |
|  |  |  |  | z-score | 66.6745 | 2.9261 |  |  |  |  |  |  |  |
|  |  | D | Naïve Bayes | Residual | 71.3421 | 4.4605 |  |  |  |  |  |  |  |
|  |  |  |  | z-score | 72.4130 | 3.5032 |  |  |  |  |  |  |  |
|  |  |  | KNN | Residual | 66.3704 | 5.0504 |  |  |  |  |  |  |  |
|  |  |  |  | z-score | 75.5 | 3.1630 |  |  |  |  |  |  |  |
|  |  |  | SVM | Residual | 0 | 0 |  |  |  |  |  |  |  |
|  |  |  |  | z-score | 73.2463 | 4.0171 |  |  |  |  |  |  |  |
|  |  |  | Logistic | Residual | 56.5912 | 5.2079 |  |  |  |  |  |  |  |
|  |  |  |  | z-score | 57.7394 | 5.8033 |  |  |  |  |  |  |  |
|  |  |  | RUSBoost | Residual | 69.0847 | 3.3470 |  |  |  |  |  |  |  |
|  |  |  |  | z-score | 69.2958 | 3.1485 |  |  |  |  |  |  |  |

**Supplementary Table 4:** Comparative analysis of classifier models and feature selection algorithms using different normalization techniques (see **Fig. 4** for complementary information) for the imbalanced analysis for OASIS-3 (age 60-86) dataset.

| Dataset | Classification | Features selection group | Classifier model | Harmonization | Mean (%) | Standard deviation | Adjustment for multiple comparison: Bonferroni | | | Adjustment for multiple comparison: Benjamini-Hochberg | | | |
| --- | --- | --- | --- | --- | --- | --- | --- | --- | --- | --- | --- | --- | --- |
|  |  |  |  |  |  |  | p-value (**Displaying only statistically significant p-values**) | 95% confidence interval | Pairwise comparison | Hypothesis ID | Raw p-value | adj. p-value | Pairwise comparison |
| OASIS-3 (age 60-86) | Accuracy | A | Naïve Bayes | Residual | 68.5645 | 2.4772 | < 0.001 | [-0.0193, -0.0060] | A < D | H3 | < 0.001 | 0.0083 | A < D |
|  |  |  |  | z-score | 69.8170 | 0.8393 | 0.002 | [-0.0158, -0.0025] | B < C | H4 | < 0.001 | 0.0167 | B < C |
|  |  |  | KNN | Residual | 59.7688 | 2.2880 | < 0.001 | [-0.0237, -0.0105] | B < D | H5 | < 0.001 | 0.025 | B < D |
|  |  |  |  | z-score | 66.3295 | 1.7311 | 0.011 | [-0.0146, 0.0013] | C < D | H6 | 0.002 | 0.033 | C < D |
|  |  |  | SVM | Residual | 79.5761 | 0 | < 0.001 | [0.0469, 0.0627] | Naïve Bayes > KNN | H1 | < 0.001 | 0.005 | Naïve Bayes > KNN |
|  |  |  |  | z-score | 78.8921 | 0.5173 | < 0.001 | [-0.1098, -0.0941] | Naïve Bayes < SVM | H2 | < 0.001 | 0.01 | Naïve Bayes < SVM |
|  |  |  | Logistic | Residual | 55.1252 | 0.1065 | < 0.001 | [0.0932, 0.1089] | Naïve Bayes > Logistic | H3 | < 0.001 | 0.015 | Naïve Bayes > Logistic |
|  |  |  |  | z-score | 61.8015 | 6.7768 | < 0.001 | [-0.0630, -0.0472] | Naïve Bayes < RUSBoost | H4 | < 0.001 | 0.02 | Naïve Bayes < RUSBoost |
|  |  |  | RUSBoost | Residual | 74.5472 | 0.8546 | < 0.001 | [-0.1646, -0.1489] | KNN < SVM | H5 | < 0.001 | 0.025 | KNN < SVM |
|  |  |  |  | z-score | 73.8247 | 1.0814 | < 0.001 | [0.0384, 0.0541] | KNN > Logistic | H6 | < 0.001 | 0.03 | KNN > Logistic |
|  |  | B | Naïve Bayes | Residual | 61.5703 | 2.2044 | < 0.001 | [-0.1178, -0.1020] | KNN < RUSBoost | H7 | < 0.001 | 0.035 | KNN < RUSBoost |
|  |  |  |  | z-score | 71.4836 | 1.0664 | < 0.001 | [0.1951, 0.2109] | SVM > Logistic | H8 | < 0.001 | 0.04 | SVM > Logistic |
|  |  |  | KNN | Residual | 60.4721 | 1.7214 | < 0.001 | [0.0390, 0.0547] | SVM > RUSBoost | H9 | < 0.001 | 0.045 | SVM > RUSBoost |
|  |  |  |  | z-score | 64.7592 | 1.7221 | < 0.001 | [-0.1640, 0.1483] | Logistic < RUSBoost | H10 | < 0.001 | 0.05 | Logistic < RUSBoost |
|  |  |  | SVM | Residual | 79.5761 | 0 | < 0.001 | [-0.031, -0.024] | Residual < Z-score | H1 | < 0.001 | 0.05 | Residual < Z-score |
|  |  |  |  | z-score | 79.3642 | 0.3746 |  |  |  |  |  |  |  |
|  |  |  | Logistic | Residual | 55.1252 | 0.1518 |  |  |  |  |  |  |  |
|  |  |  |  | z-score | 63.5260 | 7.4813 |  |  |  |  |  |  |  |
|  |  |  | RUSBoost | Residual | 74.5568 | 1.1967 |  |  |  |  |  |  |  |
|  |  |  |  | z-score | 73.3237 | 1.0011 |  |  |  |  |  |  |  |
|  |  | C | Naïve Bayes | Residual | 66.9075 | 2.1717 |  |  |  |  |  |  |  |
|  |  |  |  | z-score | 72.2929 | 1.4985 |  |  |  |  |  |  |  |
|  |  |  | KNN | Residual | 62.2929 | 2.5623 |  |  |  |  |  |  |  |
|  |  |  |  | z-score | 64.0267 | 1.8333 |  |  |  |  |  |  |  |
|  |  |  | SVM | Residual | 79.2389 | 1.1388 |  |  |  |  |  |  |  |
|  |  |  |  | z-score | 78.9499 | 0.6121 |  |  |  |  |  |  |  |
|  |  |  | Logistic | Residual | 55.1734 | 0.1686 |  |  |  |  |  |  |  |
|  |  |  |  | z-score | 63.2274 | 6.5916 |  |  |  |  |  |  |  |
|  |  |  | RUSBoost | Residual | 75.6936 | 1.0066 |  |  |  |  |  |  |  |
|  |  |  |  | z-score | 75.1156 | 1.0976 |  |  |  |  |  |  |  |
|  |  | D | Naïve Bayes | Residual | 72.2447 | 1.5672 |  |  |  |  |  |  |  |
|  |  |  |  | z-score | 70.0771 | 1.0406 |  |  |  |  |  |  |  |
|  |  |  | KNN | Residual | 65.2216 | 2.0174 |  |  |  |  |  |  |  |
|  |  |  |  | z-score | 66.2620 | 1.7334 |  |  |  |  |  |  |  |
|  |  |  | SVM | Residual | 79.5761 | 0 |  |  |  |  |  |  |  |
|  |  |  |  | z-score | 79.3545 | 0.3204 |  |  |  |  |  |  |  |
|  |  |  | Logistic | Residual | 55.2023 | 1.5938 |  |  |  |  |  |  |  |
|  |  |  |  | z-score | 62.9480 | 7.3766 |  |  |  |  |  |  |  |
|  |  |  | RUSBoost | Residual | 75.1445 | 1.0385 |  |  |  |  |  |  |  |
|  |  |  |  | z-score | 74.8459 | 0.9654 |  |  |  |  |  |  |  |
|  | F1 | A | Naïve Bayes | Residual | 40.2892 | 1.8216 | < 0.001 | [0.0045, 0.0171] | A > B | H1 | < 0.001 | 0.0083 | A > B |
|  |  |  |  | z-score | 40.7772 | 1.1797 | < 0.001 | [-0.0226, -0.0100] | B < C | H4 | < 0.001 | 0.0167 | B < C |
|  |  |  | KNN | Residual | 38.8089 | 2.3038 | < 0.001 | [-0.0180, -0.0054] | B < D | H5 | < 0.001 | 0.025 | B < D |
|  |  |  |  | z-score | 38.5943 | 2.2652 | < 0.001 | [0.0054, 0.0203] | Naïve Bayes > KNN | H2 | 0.025 | 0.033 | A < C |
|  |  |  | SVM | Residual | 0 | 0 | < 0.001 | [0.3668, 0.3817] | Naïve Bayes > SVM | H1 | < 0.001 |  | Naïve Bayes > KNN |
|  |  |  |  | z-score | 3.4872 | 3.1793 | < 0.001 | [0.0534, 0.0683] | Naïve Bayes > Logistic | H2 | < 0.001 |  | Naïve Bayes > SVM |
|  |  |  | Logistic | Residual | 40.3890 | 0.1395 | < 0.001 | [0.3539, 0.3688] | KNN > SVM | H3 | < 0.001 |  | Naïve Bayes > Logistic |
|  |  |  |  | z-score | 27.7672 | 3.7698 | < 0.001 | [0.0405, 0.0554] | KNN > Logistic | H5 | < 0.001 |  | KNN > SVM |
|  |  |  | RUSBoost | Residual | 40.5314 | 1.9544 | < 0.001 | [-0.0229, -0.0079] | KNN < RUSBoost | H6 | < 0.001 |  | KNN > Logistic |
|  |  |  |  | z-score | 40.5110 | 1.8760 | < 0.001 | [-0.3209, -0.3059] | SVM < Logistic | H7 | < 0.001 |  | KNN < RUSBoost |
|  |  | B | Naïve Bayes | Residual | 36.7397 | 1.2243 | < 0.001 | [-0.3842, -0.3693] | SVM < RUSBoost | H8 | < 0.001 |  | SVM < Logistic |
|  |  |  |  | z-score | 39.4623 | 1.7538 | < 0.001 | [-0.0708, -0.0559] | Logistic < RUSBoost | H9 | < 0.001 |  | SVM < RUSBoost |
|  |  |  | KNN | Residual | 38.8543 | 2.4859 | < 0.001 | [0.014, 0.021] | Residual > Z-score | H10 | < 0.001 |  | Logistic < RUSBoost |
|  |  |  |  | z-score | 37.3813 | 1.8011 |  |  |  | H1 | < 0.001 | 0.05 | Residual > Z-score |
|  |  |  | SVM | Residual | 0 | 0 |  |  |  |  |  |  |  |
|  |  |  |  | z-score | 1.2742 | 1.6602 |  |  |  |  |  |  |  |
|  |  |  | Logistic | Residual | 40.3279 | 0.2145 |  |  |  |  |  |  |  |
|  |  |  |  | z-score | 27.6837 | 3.4944 |  |  |  |  |  |  |  |
|  |  |  | RUSBoost | Residual | 39.1837 | 2.4782 |  |  |  |  |  |  |  |
|  |  |  |  | z-score | 39.4522 | 1.8545 |  |  |  |  |  |  |  |
|  |  | C | Naïve Bayes | Residual | 38.1505 | 1.6428 |  |  |  |  |  |  |  |
|  |  |  |  | z-score | 40.6422 | 1.6719 |  |  |  |  |  |  |  |
|  |  |  | KNN | Residual | 37.6445 | 2.7208 |  |  |  |  |  |  |  |
|  |  |  |  | z-score | 37.4457 | 1.8359 |  |  |  |  |  |  |  |
|  |  |  | SVM | Residual | 3.5272 | 3.5586 |  |  |  |  |  |  |  |
|  |  |  |  | z-score | 8.8575 | 6.3667 |  |  |  |  |  |  |  |
|  |  |  | Logistic | Residual | 40.4303 | 0.1320 |  |  |  |  |  |  |  |
|  |  |  |  | z-score | 26.7457 | 4.0245 |  |  |  |  |  |  |  |
|  |  |  | RUSBoost | Residual | 41.9387 | 2.3561 |  |  |  |  |  |  |  |
|  |  |  |  | z-score | 41.2596 | 2.0285 |  |  |  |  |  |  |  |
|  |  | D | Naïve Bayes | Residual | 42.5352 | 1.7050 |  |  |  |  |  |  |  |
|  |  |  |  | z-score | 40.7284 | 1.3597 |  |  |  |  |  |  |  |
|  |  |  | KNN | Residual | 39.0854 | 1.8219 |  |  |  |  |  |  |  |
|  |  |  |  | z-score | 41.2112 | 1.7956 |  |  |  |  |  |  |  |
|  |  |  | SVM | Residual | 0 | 0 |  |  |  |  |  |  |  |
|  |  |  |  | z-score | 2.7818 | 3.5328 |  |  |  |  |  |  |  |
|  |  |  | Logistic | Residual | 40.4152 | 0.1777 |  |  |  |  |  |  |  |
|  |  |  |  | z-score | 26.8894 | 4.8358 |  |  |  |  |  |  |  |
|  |  |  | RUSBoost | Residual | 38.9842 | 2.6277 |  |  |  |  |  |  |  |
|  |  |  |  | z-score | 39.4700 | 1.9998 |  |  |  |  |  |  |  |
|  | MCCʹ | A | Naïve Bayes | Residual | 60.6877 | 1.4722 | < 0.001 | [-0.062, -0.035] | A < C | H2 | < 0.001 | 0.0083 | A < C |
|  |  |  |  | z-score | 61.1936 | 0.7764 | < 0.001 | [-0.075, -0.048] | B < C | H4 | < 0.001 | 0.0167 | B < C |
|  |  |  | KNN | Residual | 58.7492 | 1.8826 | < 0.001 | [0.038, 0.066] | C > D | H6 | < 0.001 | 0.025 | C > D |
|  |  |  |  | z-score | 59.2975 | 1.6312 | < 0.001 | [0.292, 0.325] | Naïve Bayes > SVM | H1 | 0.014 | 0.033 | A > B |
|  |  |  | SVM | Residual | 0 | 0 | < 0.001 | [0.028, 0.060] | Naïve Bayes > Logistic | H2 | < 0.001 | 0.005 | Naibe Bayes > SVM |
|  |  |  |  | z-score | 50.6073 | 2.1925 | < 0.001 | [0.276, 0.309] | KNN > SVM | H3 | < 0.001 | 0.01 | Naïve Bayes > Logistic |
|  |  |  | Logistic | Residual | 59.9659 | 0.1309 | < 0.001 | [0.012, 0.044] | KNN > Logistic | H5 | < 0.001 | 0.015 | KNN > SVM |
|  |  |  |  | z-score | 52.3186 | 1.9531 | < 0.001 | [-0.047, -0.015] | KNN < RUSBoost | H6 | < 0.001 | 0.02 | KNN > Logistic |
|  |  |  | RUSBoost | Residual | 62.2228 | 1.1789 | < 0.001 | [-0.281, -0.248] | SVM < Logitic | H7 | < 0.001 | 0.025 | KNN < RUSBoost |
|  |  |  |  | z-score | 61.9795 | 1.2299 | < 0.001 | [-0.340, -307] | SVM < RUSBoost | H8 | < 0.001 | 0.03 | SVM < Logistic |
|  |  | B | Naïve Bayes | Residual | 57.4068 | 1.0191 | < 0.001 | [-0.075, -0.043] | Logistic < RUSBoost | H9 | < 0.001 | 0.035 | SVM < RUSBoost |
|  |  |  |  | z-score | 60.8060 | 1.0709 | < 0.001 | [-0.064, -0.050] | Residual < Z-score | H10 | < 0.001 | 0.04 | Logistic < RUSBoost |
|  |  |  | KNN | Residual | 58.8325 | 1.9804 |  |  |  | H1 | 0.006 | 0.045 | Nave Bayes > KNN |
|  |  |  |  | z-score | 58.2795 | 1.3272 |  |  |  | H4 | 0.010 | 0.05 | Naïve Bayes < RUSBoost |
|  |  |  | SVM | Residual | 0 | 0 |  |  |  | H1 | < 0.01 | 0.05 | Residual < Z-score |
|  |  |  |  | z-score | 43.2712 | 18.8271 |  |  |  |  |  |  |  |
|  |  |  | Logistic | Residual | 59.9076 | 0.2012 |  |  |  |  |  |  |  |
|  |  |  |  | z-score | 52.8537 | 2.1486 |  |  |  |  |  |  |  |
|  |  |  | RUSBoost | Residual | 61.5705 | 1.5664 |  |  |  |  |  |  |  |
|  |  |  |  | z-score | 61.2843 | 1.2171 |  |  |  |  |  |  |  |
|  |  | C | Naïve Bayes | Residual | 59.1189 | 1.2407 |  |  |  |  |  |  |  |
|  |  |  |  | z-score | 61.6382 | 1.1850 |  |  |  |  |  |  |  |
|  |  |  | KNN | Residual | 58.1054 | 2.1207 |  |  |  |  |  |  |  |
|  |  |  |  | z-score | 58.2028 | 1.4118 |  |  |  |  |  |  |  |
|  |  |  | SVM | Residual | 49.4829 | 11.9440 |  |  |  |  |  |  |  |
|  |  |  |  | z-score | 50.8442 | 12.2602 |  |  |  |  |  |  |  |
|  |  |  | Logistic | Residual | 60.0040 | 0.1223 |  |  |  |  |  |  |  |
|  |  |  |  | z-score | 52.1995 | 2.0986 |  |  |  |  |  |  |  |
|  |  |  | RUSBoost | Residual | 63.3101 | 1.4303 |  |  |  |  |  |  |  |
|  |  |  |  | z-score | 62.7716 | 1.3253 |  |  |  |  |  |  |  |
|  |  | D | Naïve Bayes | Residual | 62.6722 | 1.2459 |  |  |  |  |  |  |  |
|  |  |  |  | z-score | 61.2064 | 0.9460 |  |  |  |  |  |  |  |
|  |  |  | KNN | Residual | 59.4432 | 1.4282 |  |  |  |  |  |  |  |
|  |  |  |  | z-score | 60.9945 | 1.3456 |  |  |  |  |  |  |  |
|  |  |  | SVM | Residual | 0 | 0 |  |  |  |  |  |  |  |
|  |  |  |  | z-score | 43.6616 | 19.0091 |  |  |  |  |  |  |  |
|  |  |  | Logistic | Residual | 59.9890 | 0.1662 |  |  |  |  |  |  |  |
|  |  |  |  | z-score | 52.2091 | 2.4415 |  |  |  |  |  |  |  |
|  |  |  | RUSBoost | Residual | 61.6997 | 1.5805 |  |  |  |  |  |  |  |
|  |  |  |  | z-score | 61.8194 | 1.1930 |  |  |  |  |  |  |  |
|  | AROC | A | Naïve Bayes | Residual | 67.2232 | 1.4855 | 0.009 | [-0.0255, -0.0026] | A < C | H4 | < 0.001 | 0.0083 | B < C |
|  |  |  |  | z-score | 67.9178 | 1.0937 | < 0.001 | [-0.0320, -0.0092] | B < C | H2 | 0.002 | 0.0167 | A < C |
|  |  |  | KNN | Residual | 64.1155 | 1.9691 | 0.008 | [0.0027, 0.0256] | C > D | H6 | 0.002 | 0.025 | C > D |
|  |  |  |  | z-score | 63.7735 | 2.2731 | < 0.001 | [0.0288, 0.0560] | Naïve Bayes > KNN | H1 | < 0.001 | 0,005 | Naïve Bayes > KNN |
|  |  |  | SVM | Residual | 0 | 0 | < 0.001 | [0.3656, 0.3927] | Naïve Bayes > SVM | H2 | < 0.001 | 0,01 | Naïve Bayes > SVM |
|  |  |  |  | z-score | 59.2248 | 2.8784 | < 0.001 | [0.0958, 0.1230] | Naïve Bayes > Logistic | H3 | < 0.001 | 0,015 | Naïve Bayes > Logistic |
|  |  |  | Logistic | Residual | 59.6277 | 3.4603 | < 0.001 | [0.3232, 0.3503] | KNN > SVM | H5 | < 0.001 | 0,02 | KNN > SVM |
|  |  |  |  | z-score | 54.5287 | 2.3967 | < 0.001 | [0.0534, 0.0805] | KNN > Logistic | H6 | < 0.001 | 0,025 | KNN > Logistic |
|  |  |  | RUSBoost | Residual | 68.6673 | 1.0967 | < 0.001 | [-0.0466, -0.0194] | KNN < RUSBoost | H7 | < 0.001 | 0,03 | KNN < RUSBoost |
|  |  |  |  | z-score | 68.3061 | 1.1908 | < 0.001 | [-0.2833, -0.2562] | SVM < Logistic | H8 | < 0.001 | 0,035 | SVM < Logistic |
|  |  | B | Naïve Bayes | Residual | 66.5713 | 0.8977 | < 0.001 | [-0.3833, -0.3562] | SVM < RUSBoost | H9 | < 0.001 | 0,04 | SVM < RUSBoost |
|  |  |  |  | z-score | 67.5055 | 1.0227 | < 0.001 | [-0.1135, -0.0864] | Logistic < RUSBoost | H10 | < 0.001 | 0,045 | Logistic < RUSBoost |
|  |  |  | KNN | Residual | 63.3681 | 1.9988 | < 0.001 | [-0.105, -0.092] | Residual < Z-score | H1 | < 0.001 | 0.05 | Residual < Z-score |
|  |  |  |  | z-score | 62.8953 | 1.8717 |  |  |  |  |  |  |  |
|  |  |  | SVM | Residual | 0 | 0 |  |  |  |  |  |  |  |
|  |  |  |  | z-score | 55.3609 | 2.4932 |  |  |  |  |  |  |  |
|  |  |  | Logistic | Residual | 60.6272 | 2.5508 |  |  |  |  |  |  |  |
|  |  |  |  | z-score | 55.0623 | 2.7527 |  |  |  |  |  |  |  |
|  |  |  | RUSBoost | Residual | 67.7937 | 1.1148 |  |  |  |  |  |  |  |
|  |  |  |  | z-score | 67.6416 | 1.3682 |  |  |  |  |  |  |  |
|  |  | C | Naïve Bayes | Residual | 68.1437 | 1.0794 |  |  |  |  |  |  |  |
|  |  |  |  | z-score | 69.1354 | 0.9408 |  |  |  |  |  |  |  |
|  |  |  | KNN | Residual | 63.3636 | 2.2262 |  |  |  |  |  |  |  |
|  |  |  |  | z-score | 64.0967 | 1.9409 |  |  |  |  |  |  |  |
|  |  |  | SVM | Residual | 14.4219 | 25.6353 |  |  |  |  |  |  |  |
|  |  |  |  | z-score | 58.7988 | 2.0479 |  |  |  |  |  |  |  |
|  |  |  | Logistic | Residual | 60.0521 | 2.3341 |  |  |  |  |  |  |  |
|  |  |  |  | z-score | 54.6324 | 2.7014 |  |  |  |  |  |  |  |
|  |  |  | RUSBoost | Residual | 67.4480 | 1.0431 |  |  |  |  |  |  |  |
|  |  |  |  | z-score | 67.3300 | 1.0749 |  |  |  |  |  |  |  |
|  |  | D | Naïve Bayes | Residual | 70.3262 | 0.9983 |  |  |  |  |  |  |  |
|  |  |  |  | z-score | 69.8087 | 0.9064 |  |  |  |  |  |  |  |
|  |  |  | KNN | Residual | 63.9925 | 1.6694 |  |  |  |  |  |  |  |
|  |  |  |  | z-score | 67.1069 | 1.3086 |  |  |  |  |  |  |  |
|  |  |  | SVM | Residual | 0 | 0 |  |  |  |  |  |  |  |
|  |  |  |  | z-score | 55.5158 | 1.5998 |  |  |  |  |  |  |  |
|  |  |  | Logistic | Residual | 60.0638 | 2.5941 |  |  |  |  |  |  |  |
|  |  |  |  | z-score | 54.5454 | 2.8761 |  |  |  |  |  |  |  |
|  |  |  | RUSBoost | Residual | 66.0498 | 1.4774 |  |  |  |  |  |  |  |
|  |  |  |  | z-score | 65.8849 | 1.1820 |  |  |  |  |  |  |  |

**Supplementary Table 5:** Analysis of Variance for F1 score of balanced data analysis: measured for cross-validation measurements.


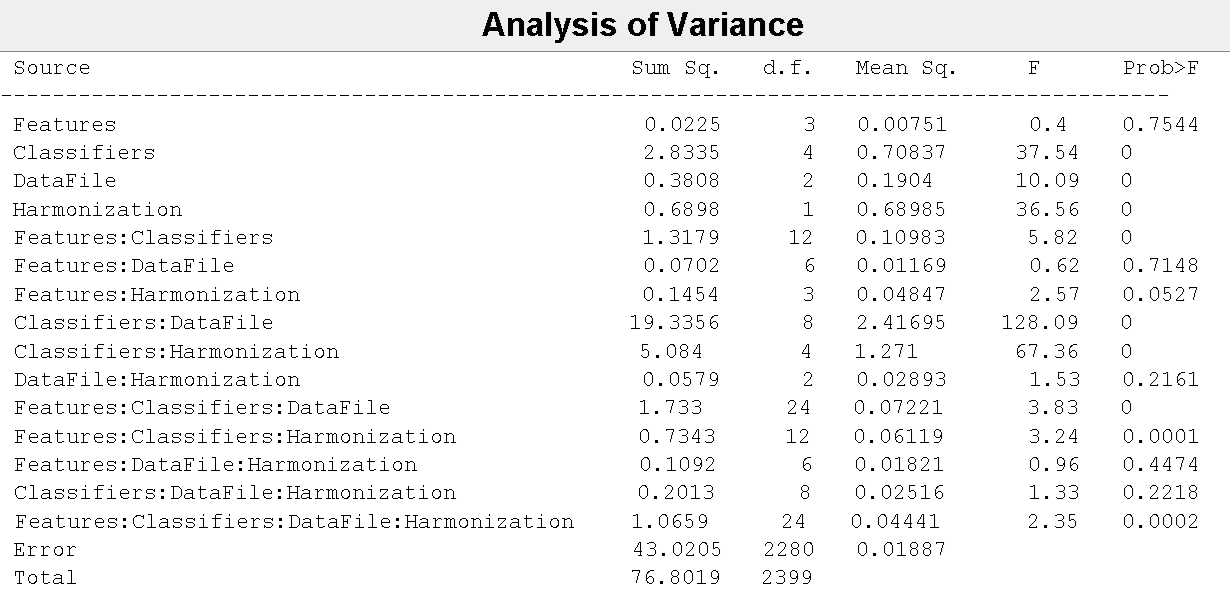


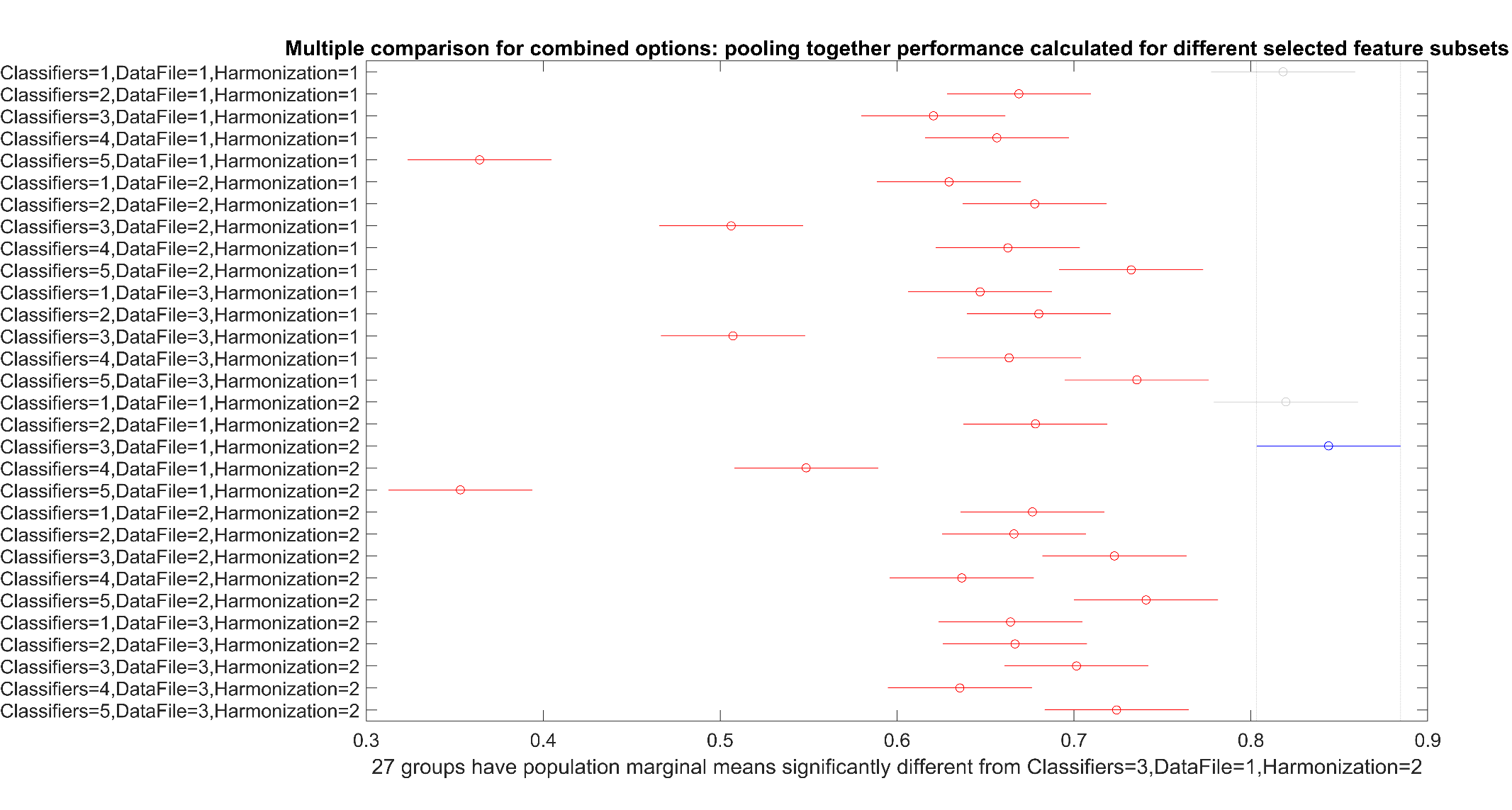


**Supplementary Figure 3:** Multiple comparisons for F1 score of balanced data analysis: measured for cross-validation (CV) measurements. Five classification models in this order: 1) Naïve Bayes, 2) KNN, 3) SVM, 4) LR, and 5) RUSBoost. Three datasets in this order: 1) ADNI, 2) OASIS-3, 3) OASIS-3 restricted to same age range as in ADNI dataset. Two harmonization procedures in this order: 1) residual and 2) z-score. The y-axis shows the label for each results entry, plotted per row, corresponding to the combination among classifier, data type, and harmonization options. The performance measure for the different feature selection subsets were pooled together in this and accompanying analyses. Best results for F1 score achieved by SVM applied for z-score harmonization of ADNI data (blue bar), which was significantly different from all the other combinations except for Naïve Bayes, which also showed “best” results for both residuals and z-score harmonization of ADNI data (grey bars).

**Supplementary Table 6:** Analysis of Variance for MCC score for balanced data analysis: measured for cross-validation measurements.


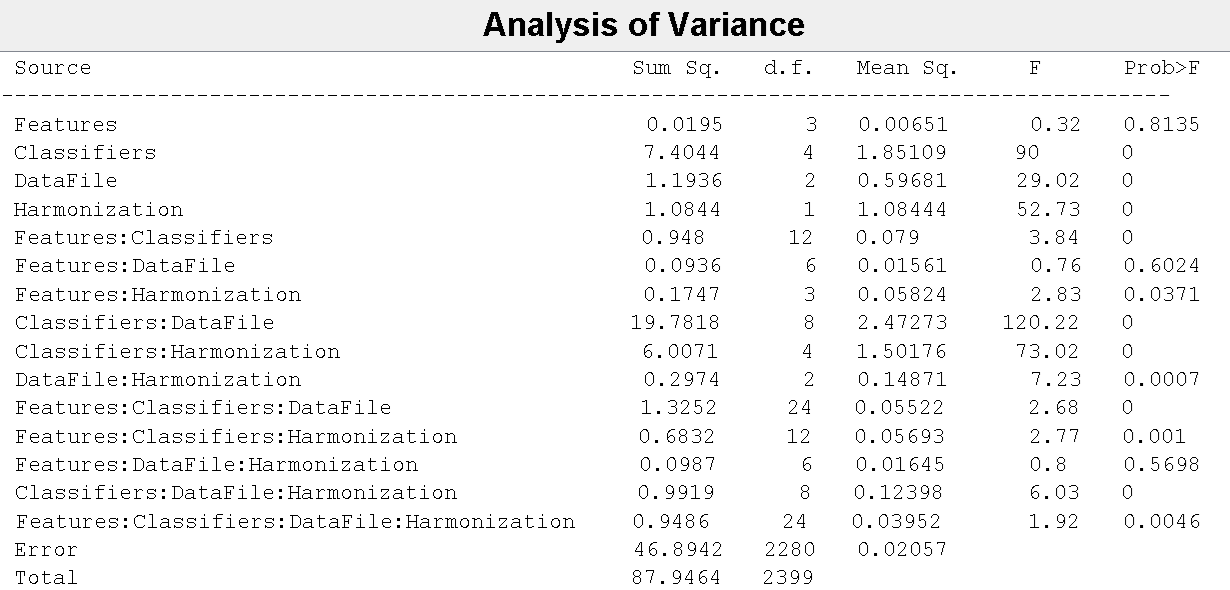


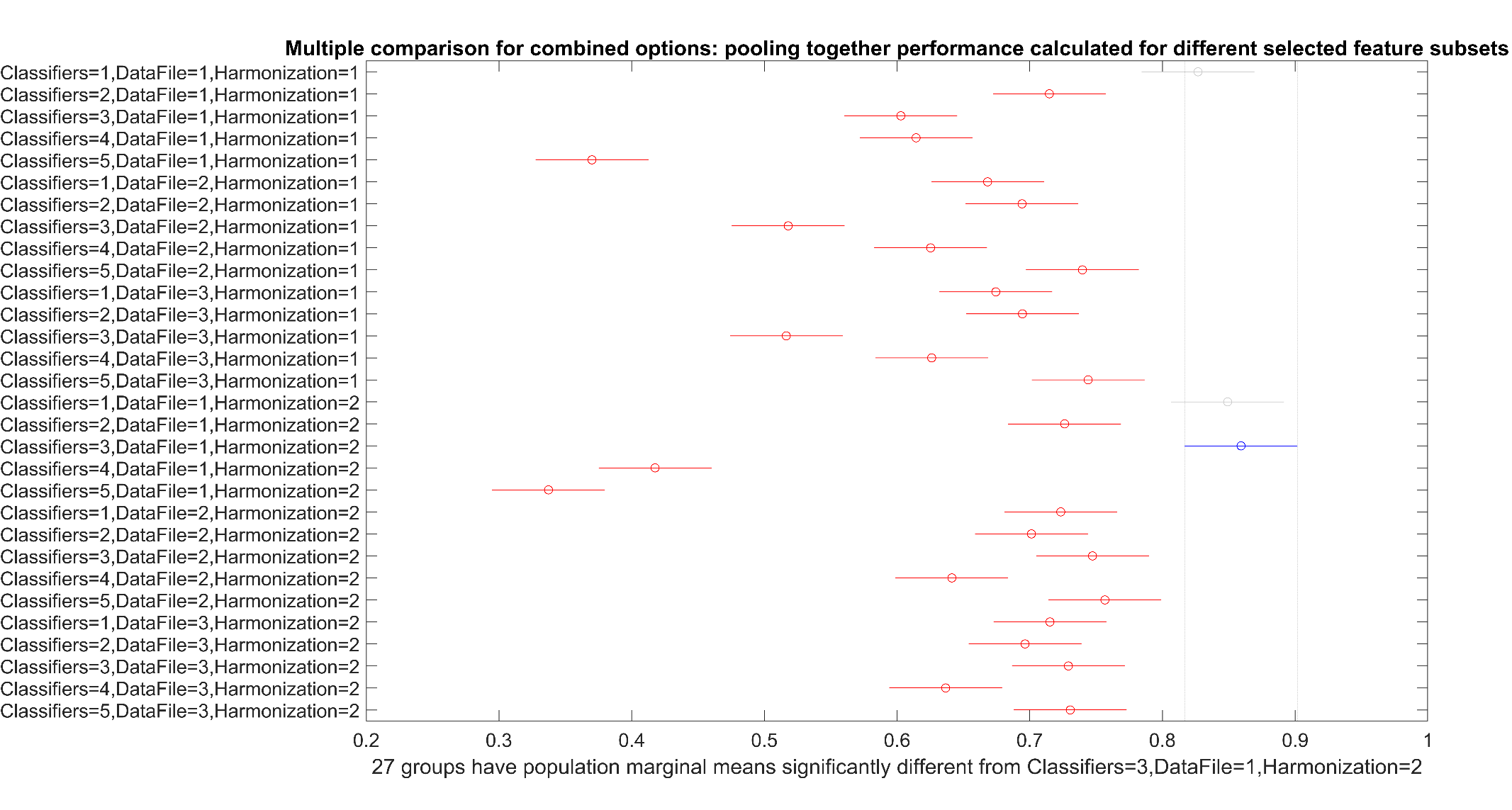


**Supplementary Figure 4:** Multiple comparisons for MCC score of balanced data analysis: measured for cross-validation (CV) measurements. Best results for MCC score achieved by SVM applied for z-score harmonization of ADNI data (blue bar), which was significantly different to the other combinations except for Naïve Bayes, which also showed “best” results for both residuals and z-score harmonization of ADNI data (grey bars). See **Supplementary Figure 3** caption for better understanding of representation.

**Supplementary Table 7:** Analysis of Variance for F1 score of imbalanced data analysis: measured for cross-validation measurements.


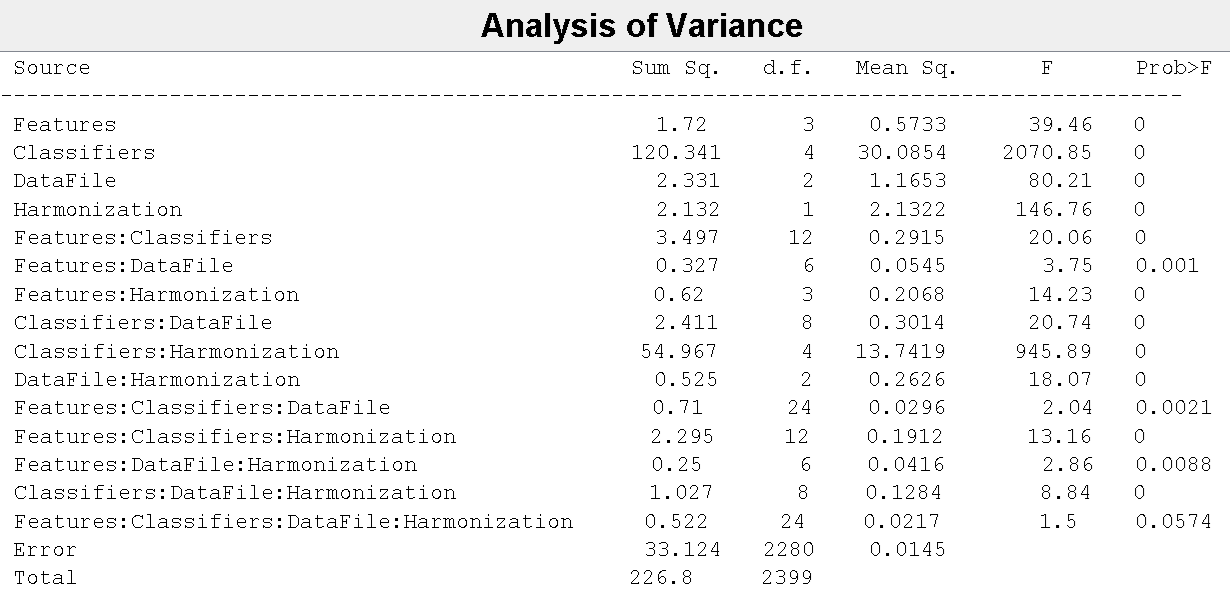


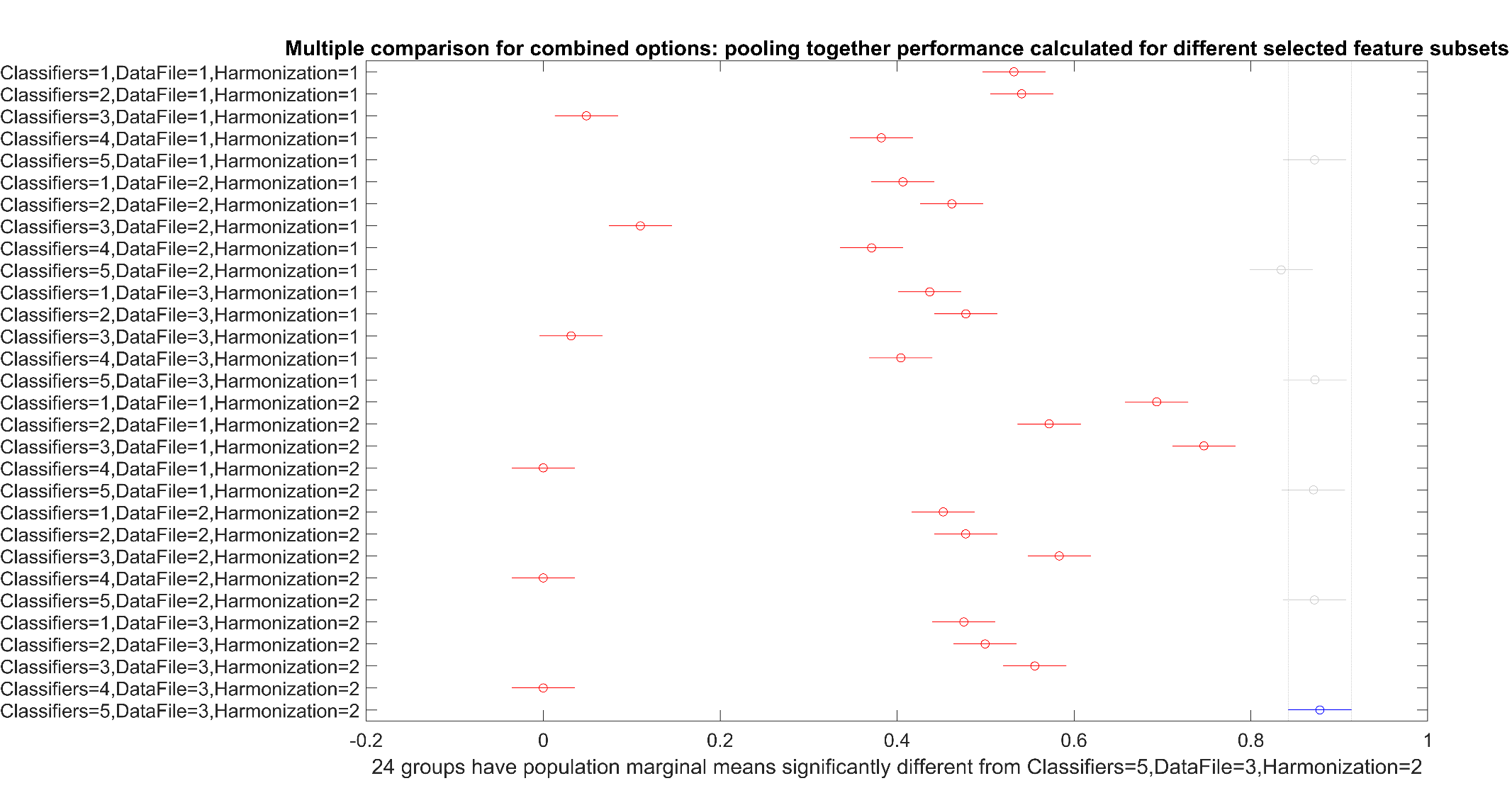


**Supplementary Figure 5:** Multiple comparisons for F1 score of imbalanced data analysis: measured for cross-validation (CV) measurements. Best results for F1 score achieved by RUSBoost for all the six combinations between data type and harmonization approach (blue and grey bars almost vertically arranged). See **Supplementary Figure 3** caption for better understanding of representation.

**Supplementary Table 8:** Analysis of Variance for MCC score of imbalanced data analysis: measured for cross-validation measurements.


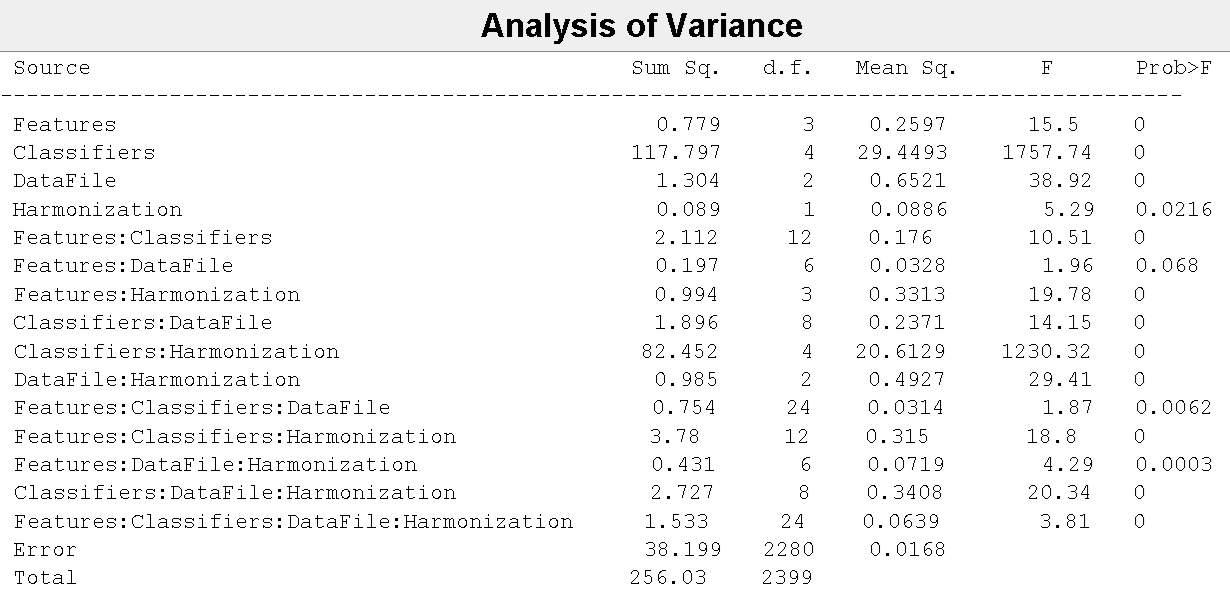


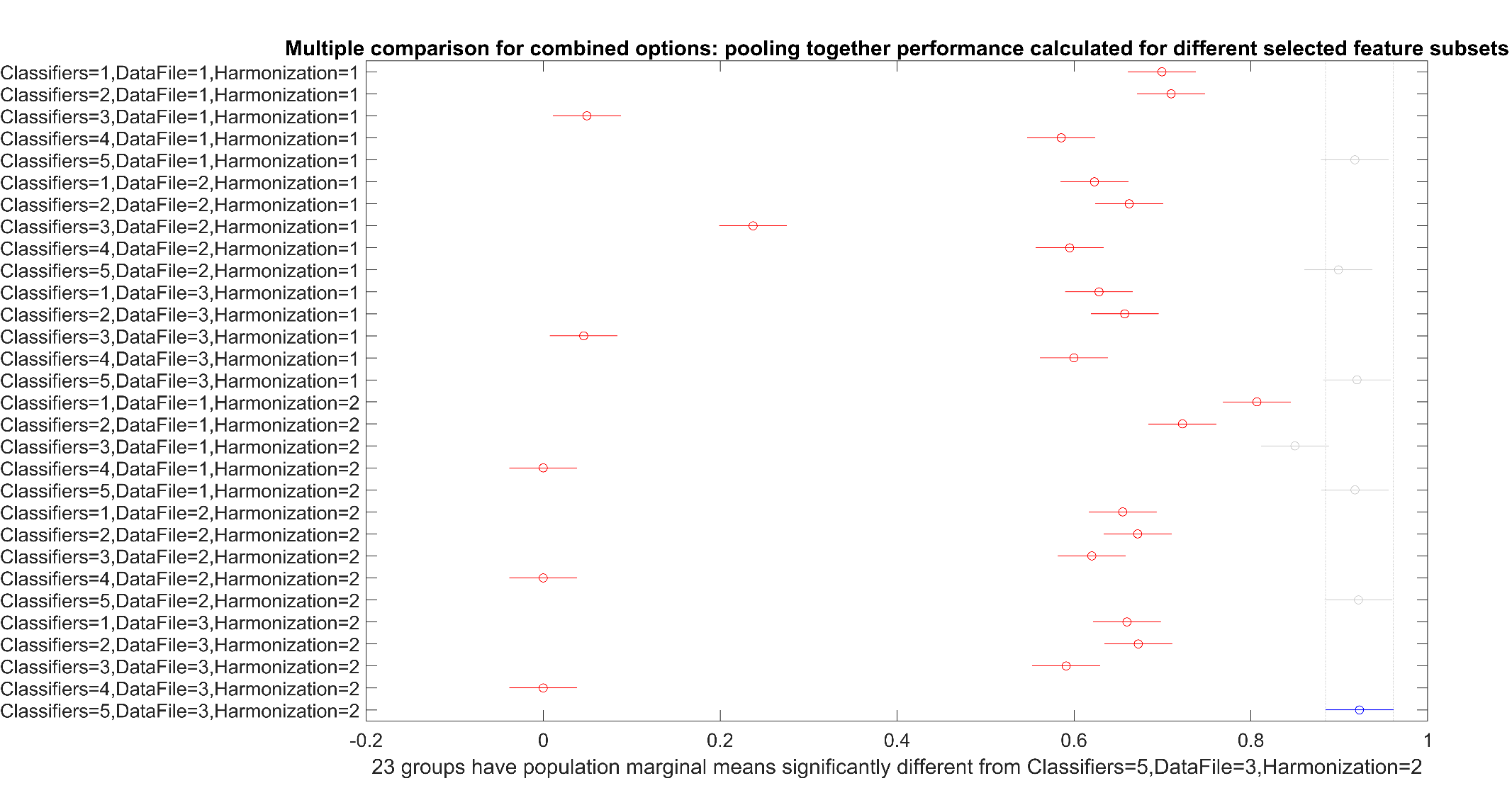


**Supplementary Figure 6:** Multiple comparisons for MCC score of imbalanced data analysis: measured for cross-validation (CV) measurements. Best results for MCC score achieved by RUSBoost for all the six combinations between data type and harmonization approach (blue and grey bars almost vertically arranged). See **Supplementary Figure 3** caption for better understanding of representation.

**Supplementary Table 9:** Analysis of Variance for F1 score of balanced data analysis: measured for nested-CV (holdout) measurements.


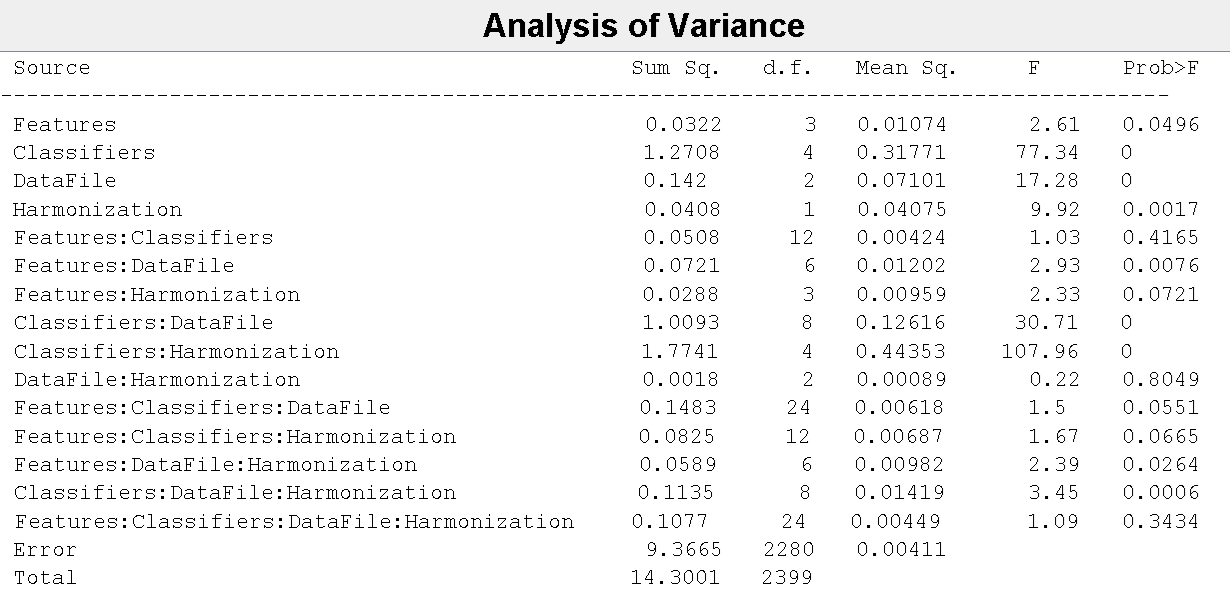


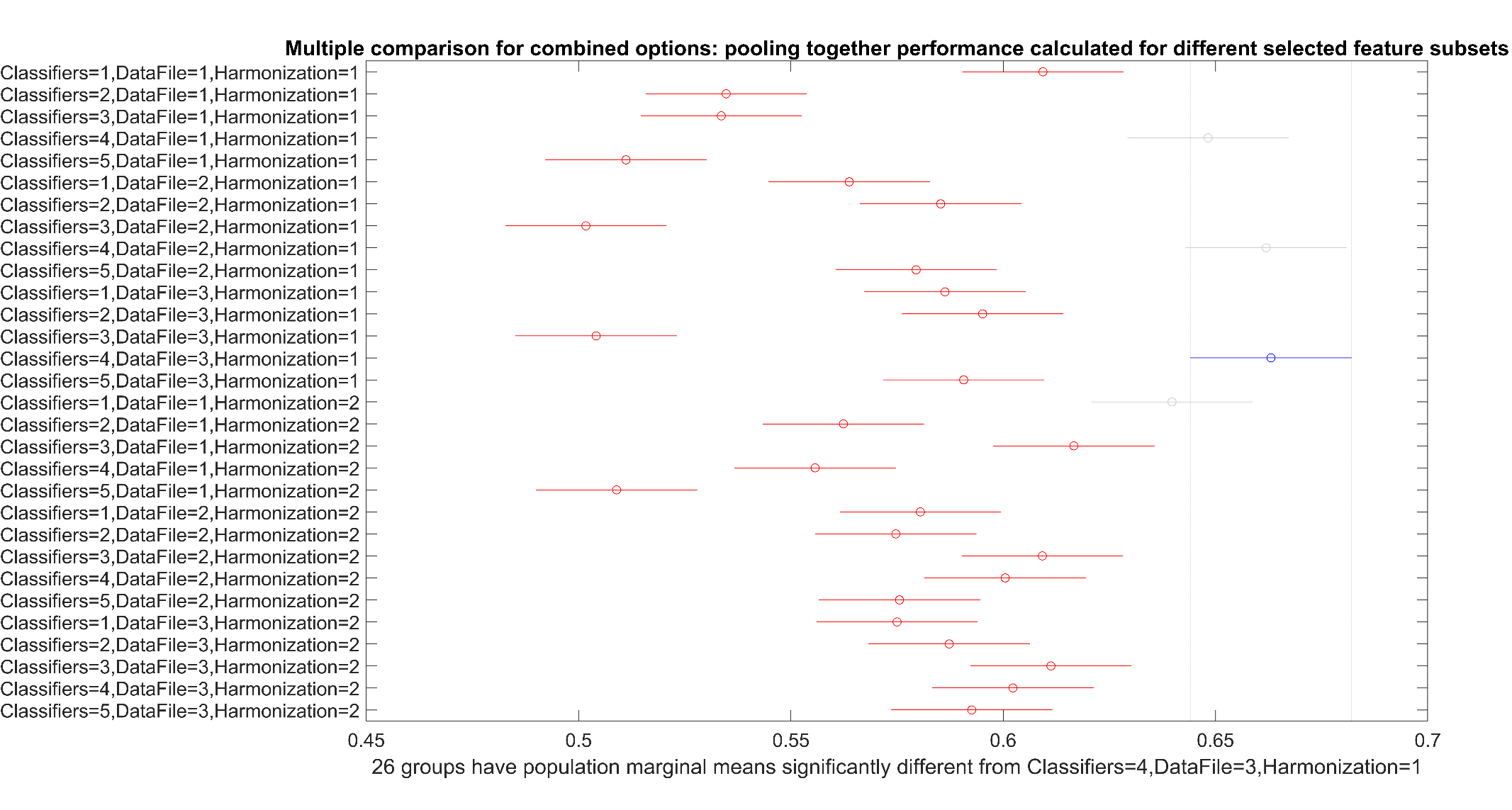


**Supplementary Figure 7:** Multiple comparisons for F1 score of balanced data analysis: measured for nested-CV (holdout) measurements. Best results for F1 score achieved by LR for all the three datasets using the residual harmonization approach (blue bar and upper grey bars), which is significantly different from all the other outcomes except from results achieved by Naïve Bayes for ADNI dataset and z-score harmonization (lower grey bar). Notice the dramatic difference between this score results (check the range of x-axis ticks) and the analogous for the CV analysis shown in **Supplementary Figure 3**, revealing the overfitting in traditional CV analysis, which is controlled by the nested-CV approach as shown with the present outcome. See **Supplementary Figure 3** caption for better understanding of representation.

**Supplementary Table 10:** Analysis of Variance for MCC score of balanced data analysis: measured for nested CV (holdout) measurements.


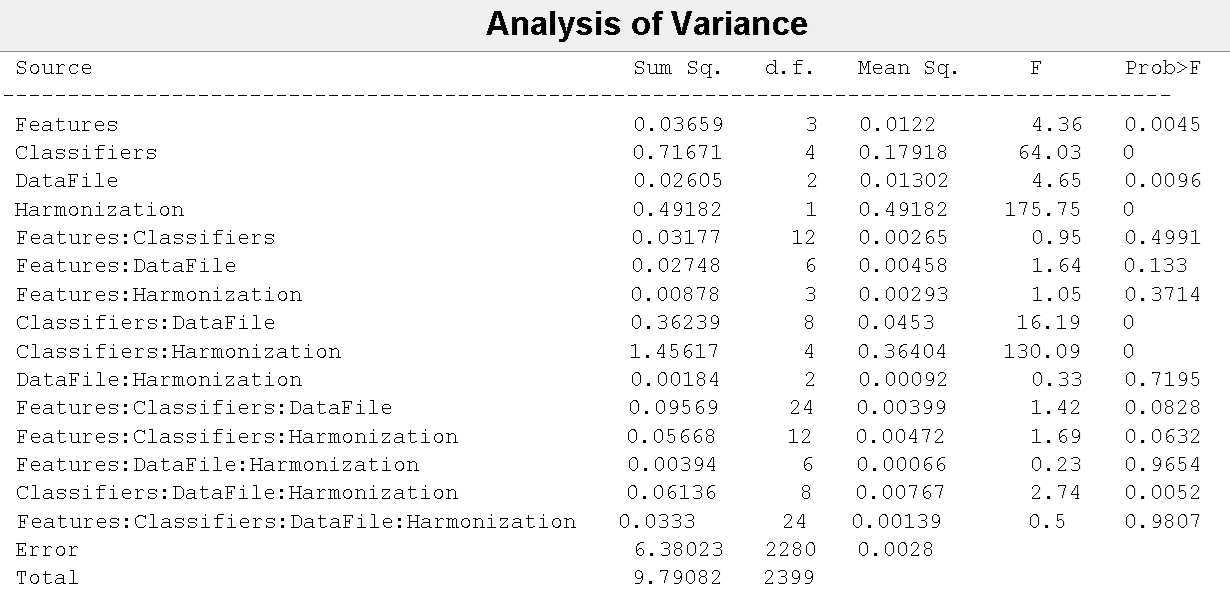


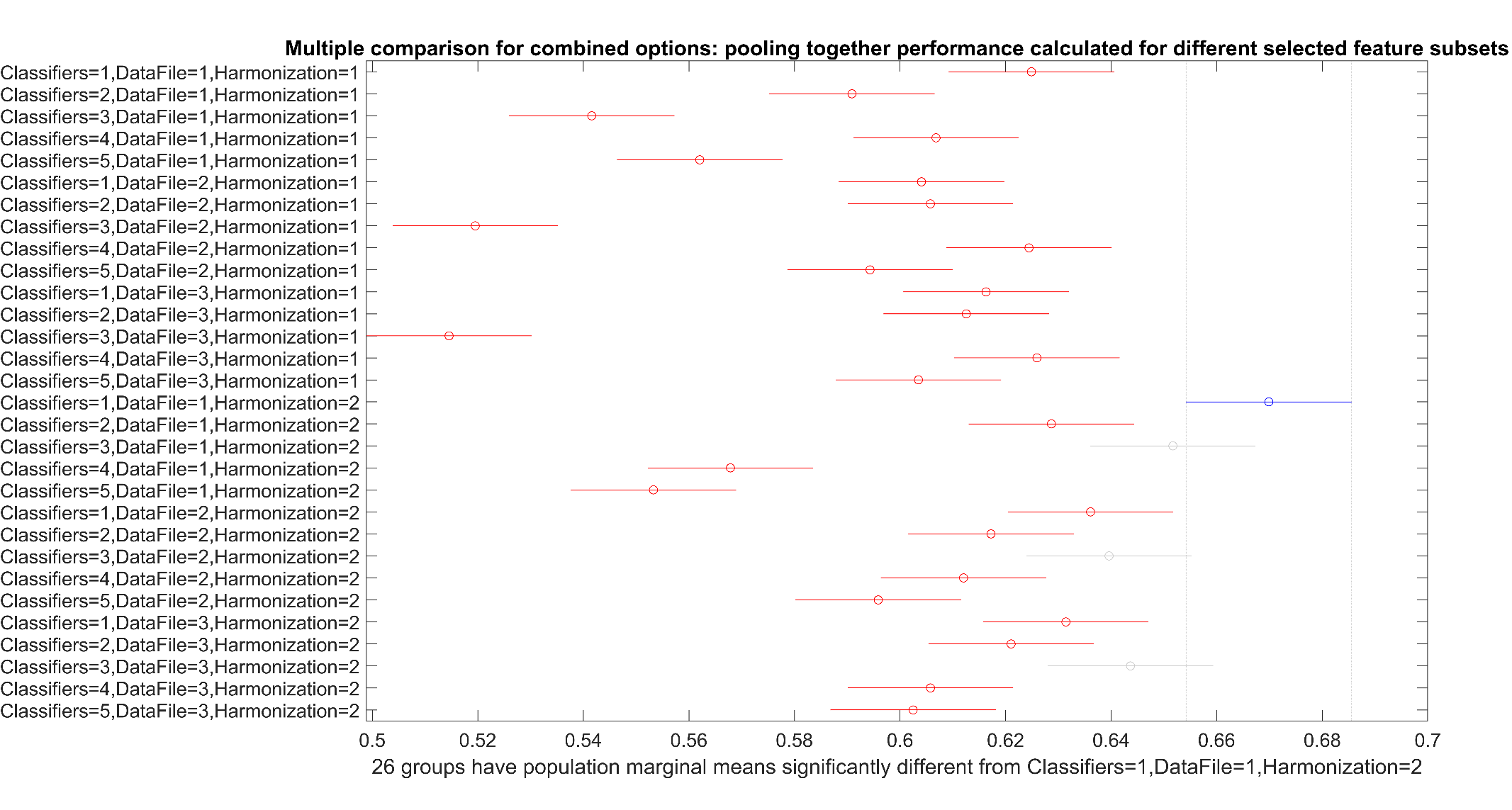


**Supplementary Figure 8:** Multiple comparisons for MCC score of balanced data analysis: measured for nested-CV (holdout) measurements. Best results for MCC score achieved by Naïve Bayes for ADNI dataset and z-score harmonization (blue bar), which is significantly different from all the other outcomes except from results achieved by SVM for all the three datasets and the z-score harmonization approach (grey bars). Notice the dramatic difference between this score results (check the range of x-axis ticks) and the analogous for the CV analysis shown in **Supplementary Figure 4**, revealing the overfitting in traditional CV analysis, which is controlled by the nested-CV approach as shown with the present outcome. See **Supplementary Figure 3** caption for better understanding of representation.

**Supplementary Table 11:** Analysis of Variance for F1 score of imbalanced data analysis: measured for nested CV (holdout) measurements.


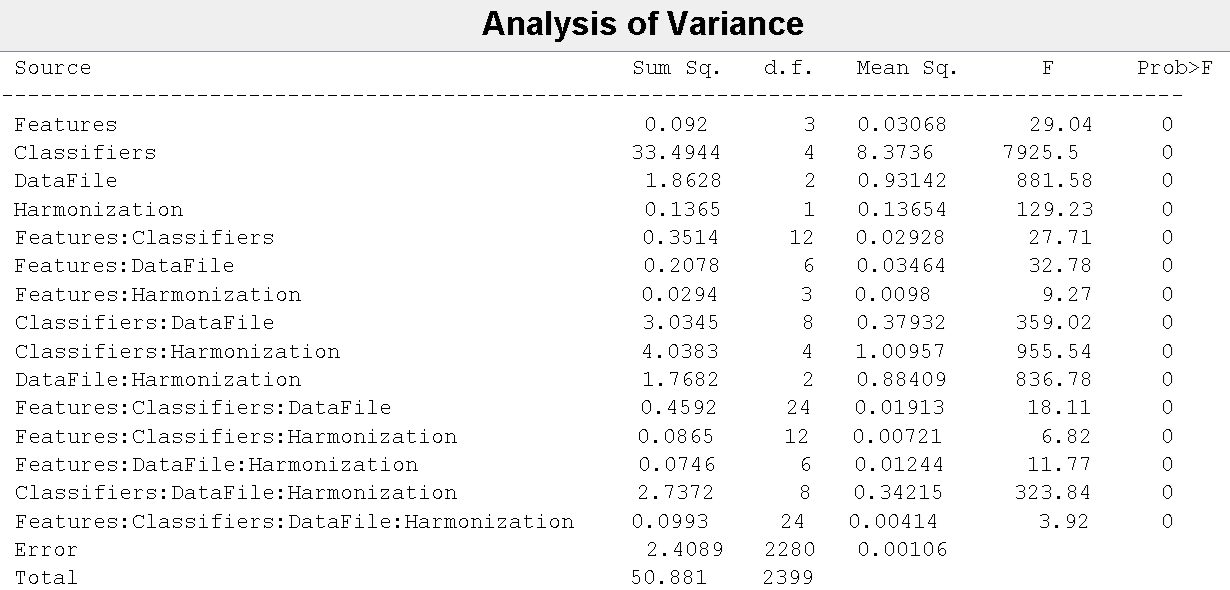


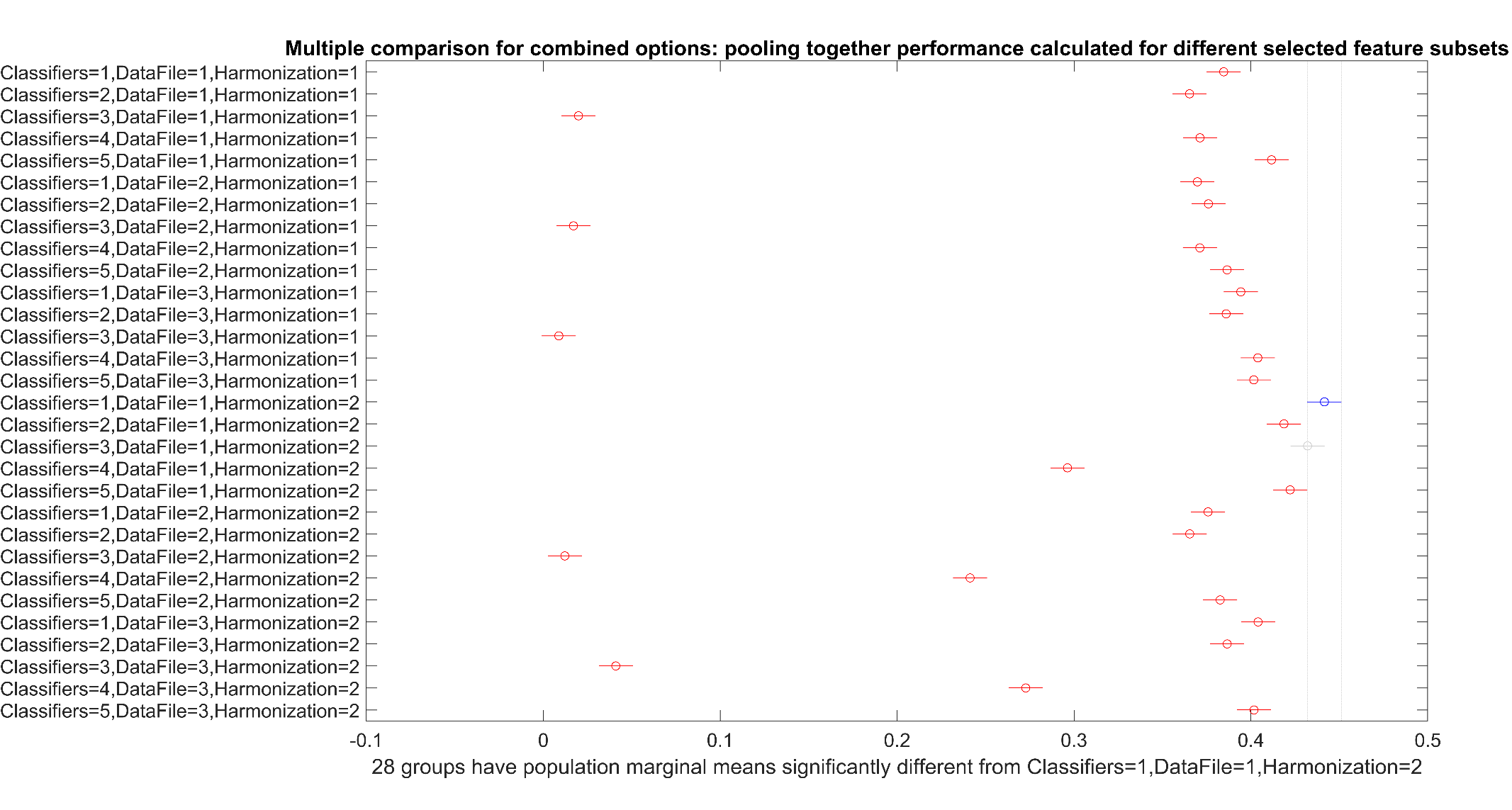


**Supplementary Figure 9:** Multiple comparisons for F1 score of imbalanced data analysis: measured for nested CV (holdout) measurements. Best results for F1 score achieved by Naïve Bayes for ADNI dataset and z-score harmonization (blue bar), which is significantly different from all the other outcomes except from results achieved by SVM for ADNI dataset and the z-score harmonization (grey bar). Notice the dramatic difference between this score results (check the range of x-axis ticks) and the analogous for the CV analysis shown in **Supplementary Figure 5**, revealing the overfitting in traditional CV analysis, which is controlled by the nested CV approach as shown with the present outcome. See **Supplementary Figure 3** caption for better understanding of representation.

**Supplementary Table 12:** Analysis of Variance for MCC score of imbalanced data analysis: measured for nested CV (holdout) measurements.


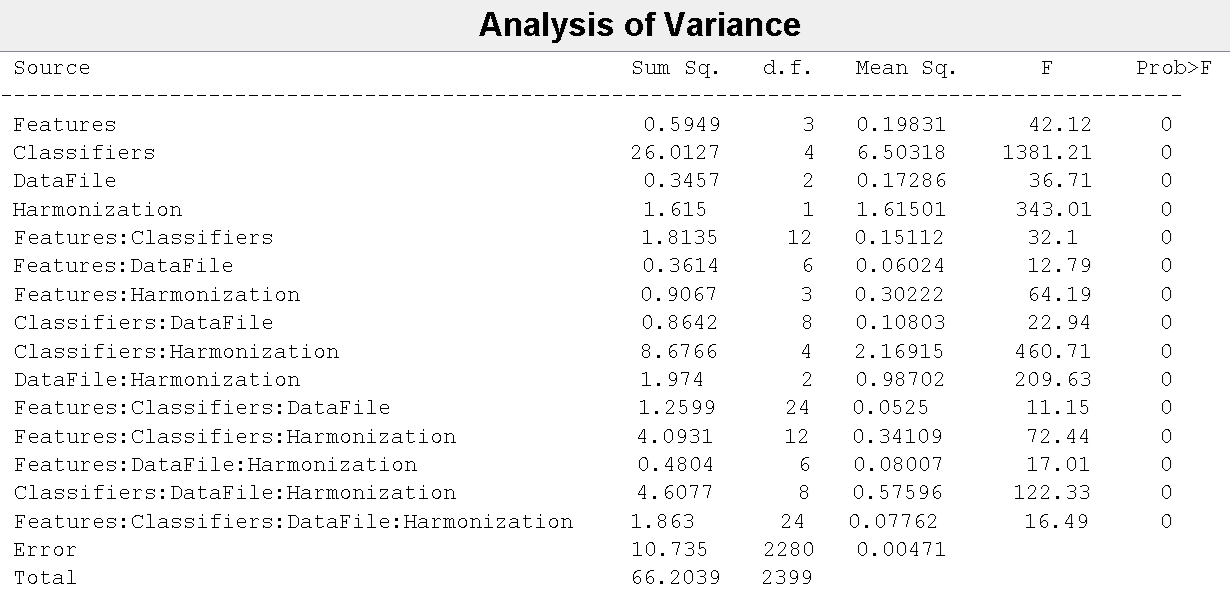


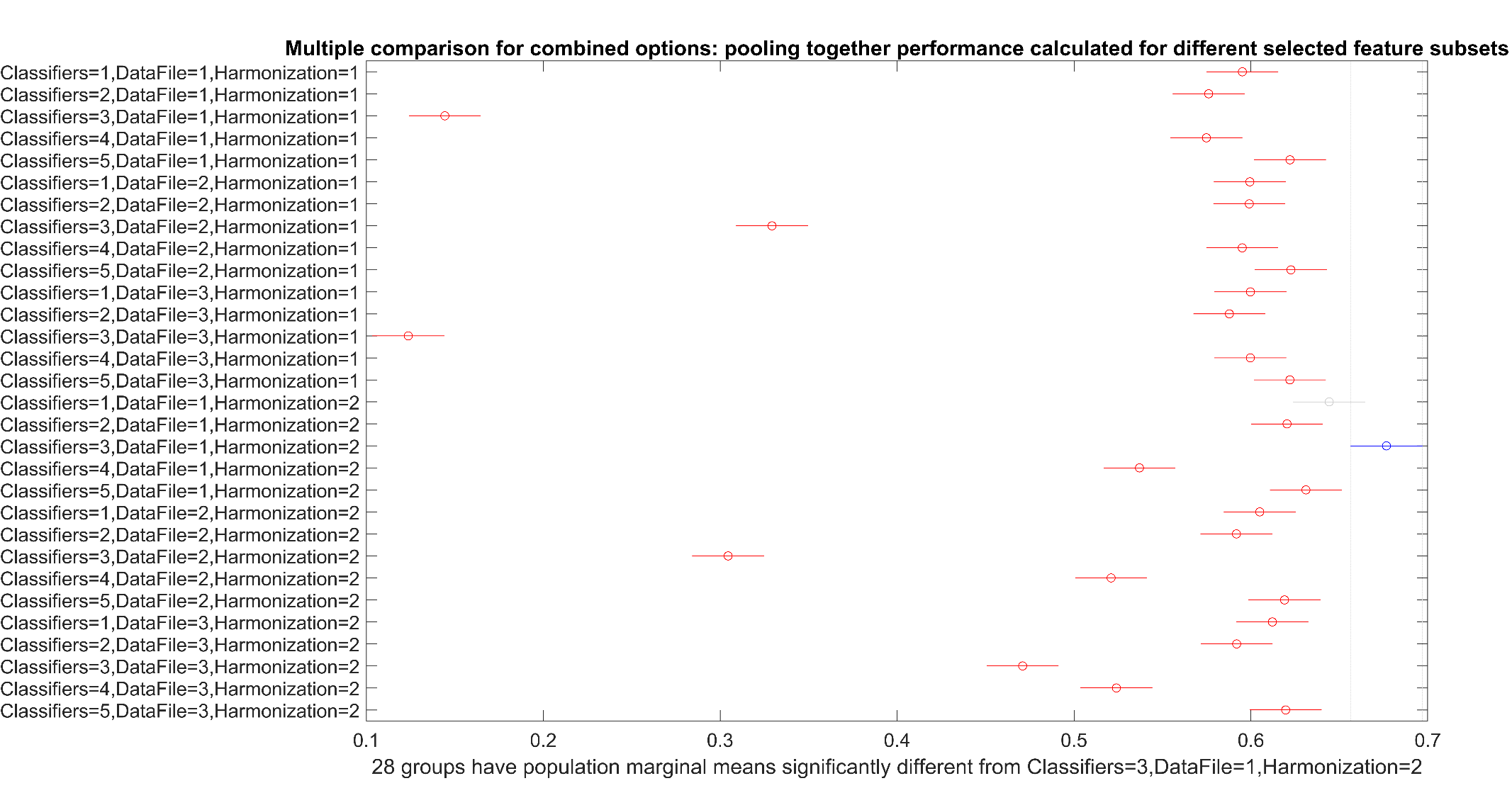


**Supplementary Figure 10:** Multiple comparisons for MCC score of imbalanced data analysis: measured for nested CV (holdout) measurements. Best results for MCC score achieved by SVM for ADNI dataset and z-score harmonization (blue bar), which is significantly different from all the other outcomes except from results achieved by Naïve Bayes for ADNI dataset and the z-score harmonization (grey bar). Notice the dramatic difference between this score results (check the range of x-axis ticks) and the analogous for the CV analysis shown in **Supplementary Figure 6**, revealing the overfitting in traditional CV analysis, which is controlled by the nested CV approach as shown with the present outcome. See **Supplementary Figure 3** caption for better understanding of representation.

**Supplementary Table 13:** Comparison of classification performance for balanced data analysis for different classification methods and feature selection criteria (see **Figure 5** caption for more information). The features selected under the four different selection criteria evaluated in our research are listed below at the left side of the table, in order correspondingly to the **subsets A-D** mentioned in the manuscript.

| **Features** | **Models** | **Dataset** | **Acc (Mean (SD)) (%)** | **AROC (Mean (SD)) (%)** | **F1**  **(Mean (SD)) (%)** | **MCC (Mean (SD)) (%)** | **Acc (Mean (SD)) (%)** | **AROC (Mean (SD)) (%)** | **F1**  **(Mean (SD)) (%)** | **MCCʹ (Mean (SD)) (%)** |
| --- | --- | --- | --- | --- | --- | --- | --- | --- | --- | --- |
|  |  |  | **residual harmonization** | | | | **z-score harmonization** | | | |
| 1. **Average score percentage (MCL app): chi-square, ReliefF, ANOVA & Kruskal Wallis (20 features)** | | | | | | | | | | |
| **brainSegVolNotVent**  **entorhinal**  **inf-Lat-Vent**  **lateral-Ventricle parahippocampal hippocampus**  **temporalpole**  **fusiform**  **lateralorbitofrontal**  **insula**  **precentral**  **isthmuscingulate**  **parsopercularis**  **posteriorcingulate**  **accumbens-area**  **rostralmiddlefrontal**  **middletemporal**  **rostralanteriorcingulate**  **amygdala**  **parsorbitalis** | **Naïve Bayes** | **ADNI**  **(age: 60-86)** | 59.69 (7.77) | 69.60 (10.78) | 57.43 (9.87) | 59.74 (7.85) | 63.75 (5.91) | 73.15 (6.31) | 60.25 (8.05) | 64.30 (6.21) |
|  |  | **OASIS-3**  **(age: 43-96)** | 59.83 (3.08) | 65.57 (4.01) | 57.53 (3.70) | 59.94 (3.10) | 62.93 (2.34) | 67.89 (2.46) | 58.36 (3.69) | 63.28 (2.36) |
|  |  | **OASIS-3**  **(age: 60-86)** | 60.73 (3.13) | 66.24 (2.14) | 58.51 (3.96) | 60.90 (3.22) | 63.00 (3.29) | 68.02 (3.18) | 57.91 (5.18) | 63.40 (3.30) |
|  | **KNN** | **ADNI**  **(age: 60-86)** | 57.29 (5.72) | 62.48 (6.34) | 51.50 (7.64) | 57.58 (5.79) | 59.79 (6.12) | 67.31 (7.40) | 52.61 (9.81) | 60.49 (6.56) |
|  |  | **OASIS-3**  **(age: 43-96)** | 59.81 (3.29) | 65.52 (3.09) | 59.33 (4.23) | 59.83 (3.29) | 61.11 (2.68) | 64.90 (3.06) | 56.93 (4.21) | 61.43 (2.82) |
|  |  | **OASIS-3**  **(age: 60-86)** | 61.06 (3.00) | 64.18 (3.66) | 59.46 (4.44) | 61.15 (2.99) | 60.57 (2.34) | 65.13 (2.99) | 57.84 (4.07) | 60.73 (2.36) |
|  | **SVM** | **ADNI**  **(age: 60-86)** | 56.04 (7.40) | 53.33 (20.67) | 54.86 (8.18) | 56.06 (7.41) | 65.83 (8.87) | 73.71 (8.56) | 62.22 (10.20) | 66.22 (9.04) |
|  |  | **OASIS-3**  **(age: 43-96)** | 52.48 (4.44) | 50.36 (12.97) | 50.94 (5.38) | 52.49 (4.46) | 63.12 (3.89) | 68.46 (3.48) | 59.75 (5.14) | 63.33 (3.91) |
|  |  | **OASIS-3**  **(age: 60-86)** | 50.09 (5.94) | 50.12 (7.64) | 49.51 (5.79) | 50.10 (5.95) | 63.84 (3.24) | 69.04 (3.37) | 61.51 (4.24) | 63.97 (3.23) |
|  | **Logistic** | **ADNI**  **(age: 60-86)** | 60.83 (3.42) | 57.92 (8.03) | 64.91 (2.07) | 61.12 (3.33) | 57.19 (9.47) | 61.69 (10.58) | 56.46 (9.36) | 57.22 (9.50) |
|  |  | **OASIS-3**  **(age: 43-96)** | 61.67 (1.67) | 58.87 (3.62) | 65.92 (1.02) | 62.04 (1.63) | 60.15 (4.52) | 65.30 (5.16) | 59.08 (4.29) | 60.18 (4.54) |
|  |  | **OASIS-3**  **(age: 60-86)** | 61.82 (2.08) | 60.67 (2.74) | 66.06 (1.29) | 62.19 (2.03) | 59.10 (3.33) | 63.24 (3.72) | 58.48 (3.13) | 59.13 (3.34) |
|  | **RUSBoost** | **ADNI**  **(age: 60-86)** | 54.69 (9.46) | 49.56 (15.28) | 47.14 (13.79) | 54.73 (9.80) | 56.46 (10.40) | 52.25 (10.00) | 52.88 (12.58) | 56.56 (10.59) |
|  |  | **OASIS-3**  **(age: 43-96)** | 58.95 (4.53) | 61.49 (4.75) | 58.13 (7.09) | 59.13 (4.62) | 59.91 (4.16) | 61.94 (4.38) | 56.79 (5.62) | 60.14 (4.23) |
|  |  | **OASIS-3**  **(age: 60-86)** | 59.22 (4.46) | 61.75 (4.87) | 58.29 (6.22) | 59.29 (4.47) | 60.57 (5.02) | 62.75 (4.42) | 60.44 (7.31) | 60.81 (5.22) |
| 1. **ReliefF (19 features)** | | | | | | | | | | |
| **entorhinal**  **fusiform**  **Inf-Lat-Vent**  **Temporalpole**  **Posteriorcingulate**  **Isthmuscingulate**  **hippocampus**  **parahippocampal**  **parsopercularis**  **Lateral-Ventricle**  **transversetemporal**  **precentral**  **insula**  **frontalpole**  **superiortemporal**  **amygdala**  **lateralorbitofrontal**  **lingual**  **BrainSegVolNotVent** | **Naïve Bayes** | **ADNI**  **(age: 60-86)** | 63.54 (9.28) | 71.10 (10.80) | 62.04 (10.54) | 63.60 (9.31) | 69.17 (6.54) | 77.73 (7.08) | 69.21 (7.90) | 69.28 (6.56) |
|  |  | **OASIS-3**  **(age: 43-96)** | 59.25 (3.31) | 65.48 (2.95) | 56.19 (3.70) | 59.47 (3.51) | 62.65 (2.44) | 68.41 (2.700) | 57.05 (5.50) | 63.13 (2.26) |
|  |  | **OASIS-3**  **(age: 60-86)** | 60.71 (3.45) | 66.43 (3.13) | 60.34 (4.29) | 60.86 (3.57) | 60.99 (2.57) | 66.62 (2.11) | 55.05 (3.73) | 61.42 (2.65) |
|  | **KNN** | **ADNI**  **(age: 60-86)** | 59.17 (9.99) | 62.67 (10.26) | 53.32 (13.56) | 59.45 (10.06) | 62.71 (5.61) | 70.06 (7.91) | 57.99 (6.96) | 63.11 (5.73) |
|  |  | **OASIS-3**  **(age: 43-96)** | 59.27 (3.15) | 62.86 (4.05) | 57.14 (5.41) | 59.37 (3.14) | 59.53 (1.56) | 63.66 (2.23) | 53.44 (4.54) | 60.01 (1.79) |
|  |  | **OASIS-3**  **(age: 60-86)** | 61.58 (4.38) | 64.40 (4.59) | 61.65 (4.99) | 61.62 (4.38) | 61.39 (2.71) | 65.45 (3.83) | 56.91 (5.46) | 61.75 (2.72) |
|  | **SVM** | **ADNI**  **(age: 60-86)** | 53.85 (6.49) | 45.04 (24.83) | 52.92 (7.11) | 53.88 (6.56) | 65.73 (7.66) | 70.67 (8.50) | 64.37 (7.90) | 65.89 (7.71) |
|  |  | **OASIS-3**  **(age: 43-96)** | 51.97 (4.58) | 50.03 (13.39) | 50.90 (4.60) | 51.97 (4.59) | 62.78 (2.12) | 67.72 (2.56) | 59.94 (3.15) | 62.93 (2.11) |
|  |  | **OASIS-3**  **(age: 60-86)** | 51.58 (5.41) | 50.53 (13.54) | 50.47 (6.14) | 51.58 (5.43) | 63.14 (3.21) | 68.84 (2.33) | 59.19 (4.40) | 63.43 (3.22) |
|  | **Logistic** | **ADNI**  **(age: 60-86)** | 60.41 (4.11) | 55.83 (7.98) | 64.81 (2.59) | 60.70 (4.11) | 55.21 (7.20) | 59.63 (9.66) | 53.66 (8.26) | 55.27 (7.27) |
|  |  | **OASIS-3**  **(age: 43-96)** | 61.73 (2.47) | 59.03 (3.17) | 65.99 (1.48) | 62.10 (2.39) | 60.94 (3.46) | 65.43 (4.01) | 59.35 (3.69) | 61.00 (3.49) |
|  |  | **OASIS-3**  **(age: 60-86)** | 62.83 (1.93) | 61.55 (3.98) | 66.68 (1.21) | 63.18 (1.87) | 60.26 (3.06) | 64.31 (3.23) | 59.71 (3.29) | 60.30 (3.07) |
|  | **RUSBoost** | **ADNI**  **(age: 60-86)** | 57.19 (7.81) | 55.52 (12.06) | 53.15 (10.19) | 57.39 (8.04) | 53.33 (9.83) | 48.88 (14.29) | 48.46 (11.21) | 53.48 (10.23) |
|  |  | **OASIS-3**  **(age: 43-96)** | 59.81 (3.17) | 61.77 (4.02) | 57.88 (7.02) | 60.07 (3.18) | 59.58 (3.90) | 61.19 (4.37) | 56.22 (6.16) | 59.58 (4.67) |
|  |  | **OASIS-3**  **(age: 60-86)** | 60.40 (4.49) | 61.99 (3.86) | 59.16 (7.03) | 60.54 (4.48) | 58.16 (3.63) | 59.69 (4.28) | 57.76 (6.80) | 58.32 (3.70) |
| 1. **Frequent feature appearances from all feature ranking** **analysis (7 features)** | | | | | | | | | | |
| **BrainSegVolNotVent**  **Inf-Lat-Vent**  **entorhinal**  **lateralorbitofrontal**  **Lateral-Ventricle**  **parahippocampal**  **hippocampus** | **Naïve Bayes** | **ADNI**  **(age: 60-86)** | 62.40 (8.51) | 69.10 (11.24) | 61.57 (9.44) | 62.44 (8.56) | 67.40 (5.16) | 74.08 (6.35) | 63.90 (6.26) | 67.83 (5.31) |
|  |  | **OASIS-3**  **(age: 43-96)** | 59.74 (3.92) | 67.30 (3.76) | 54.70 (3.81) | 60.10 (4.20) | 63.91 (3.13) | 70.14 (2.67) | 58.52 (5.51) | 64.40 (2.99) |
|  |  | **OASIS-3**  **(age: 60-86)** | 61.13 (2.90) | 67.55 (3.11) | 56.67 (3.92) | 61.58 (3.20) | 63.23 (2.59) | 68.70 (2.32) | 57.65 (5.00) | 63.73 (2.47) |
|  | **KNN** | **ADNI**  **(age: 60-86)** | 58.23 (7.14) | 63.77 (7.81) | 53.02 (9.90) | 58.47 (7.24) | 63.96 (6.05) | 70.85 (6.90) | 58.00 (9.50) | 64.68 (6.31) |
|  |  | **OASIS-3**  **(age: 43-96)** | 61.67 (3.11) | 65.36 (3.30) | 58.93 (4.01) | 61.86 (3.20) | 62.84 (2.33) | 67.18 (2.59) | 59.60 (2.92) | 63.04 (2.37) |
|  |  | **OASIS-3**  **(age: 60-86)** | 60.45 (2.91) | 64.34 (3.15) | 57.13 (3.28) | 60.64 (3.03) | 62.59 (2.83) | 66.76 (3.49) | 59.07 (3.42) | 62.85 (2.96) |
|  | **SVM** | **ADNI**  **(age: 60-86)** | 56.04 (7.85) | 57.92 (8.88) | 55.75 (7.29) | 56.06 (7.96) | 64.27 (7.16) | 72.33 (6.54) | 59.99 (8.12) | 64.69 (7.39) |
|  |  | **OASIS-3**  **(age: 43-96)** | 53.16 (4.60) | 54.82 (6.75) | 51.16 (5.74) | 53.18 (4.65) | 64.10 (3.22) | 70.34 (3.24) | 61.12 (4.34) | 64.30 (3.21) |
|  |  | **OASIS-3**  **(age: 60-86)** | 51.77 (3.71) | 53.07 (5.97) | 50.28 (4.07) | 51.78 (3.74) | 63.09 (3.39) | 68.76 (3.84) | 59.85 (4.79) | 63.59 (3.36) |
|  | **Logistic** | **ADNI**  **(age: 60-86)** | 60.42 (4.73) | 57.48 (9.00) | 64.99 (2.35) | 60.70 (4.66) | 58.96 (6.27) | 61.75 (6.58) | 57.44 (6.47) | 59.07 (6.32) |
|  |  | **OASIS-3**  **(age: 43-96)** | 62.56 (2.19) | 60.53 (3.27) | 66.50 (1.31) | 62.91 (2.13) | 61.13 (3.24) | 64.79 (4.76) | 60.43 (2.75) | 61.16 (3.25) |
|  |  | **OASIS-3**  **(age: 60-86)** | 62.26 (2.41) | 60.62 (3.72) | 66.34 (1.51) | 62.62 (2.35) | 60.78 (4.81) | 64.41 (4.41) | 60.89 (4.51) | 60.79 (4.81) |
|  | **RUSBoost** | **ADNI**  **(age: 60-86)** | 56.98 (9.15) | 56.54 (13.69) | 52.37 (12.10) | 57.16 (9.60) | 55.00 (7.45) | 52.13 (13.40) | 50.10 (9.26) | 55.13 (7.71) |
|  |  | **OASIS-3**  **(age: 43-96)** | 59.74 (3.87) | 62.22 (3.73) | 58.47 (5.16) | 59.81 (3.88) | 60.13 (4.38) | 62.25 (4.10) | 59.45 (5.65) | 60.21 (4.39) |
|  |  | **OASIS-3**  **(age: 60-86)** | 60.26 (4.82) | 62.35 (4.27) | 58.83 (6.67) | 60.49 (4.94) | 61.08 (4.68) | 62.83 (5.11) | 59.71 (5.89) | 61.21 (4.75) |
| 1. **SPSS statistical analysis’ extracted features (8 features)** | | | | | | | | | | |
| **Entorhinal**  **Inf-Lat-Vent**  **Hippocampus**  **Lateral-Ventricle**  **lateralorbitofrontal**  **BrainSegVolNotVent**  **Accumbens-area**  **middle temporal** | **Naïve Bayes** | **ADNI**  **(age: 60-86)** | 64.06 (7.40) | 69.56 (7.38) | 62.70 (7.38) | 64.17 (7.47) | 66.15 (4.37) | 73.54 (5.29) | 62.54 (4.90) | 66.53 (4.49) |
|  |  | **OASIS-3**  **(age: 43-96)** | 61.65 (3.42) | 67.67 (2.47) | 57.07 (3.58) | 62.11 (3.76) | 63.23 (2.08) | 70.11 (1.62) | 58.27 (3.26) | 63.63 (2.08) |
|  |  | **OASIS-3**  **(age: 60-86)** | 62.81 (3.16) | 69.23 (3.19) | 59.00 (2.91) | 63.18 (3.42) | 63.61 (2.27) | 69.94 (2.39) | 59.38 (3.44) | 64.02 (2.39) |
|  | **KNN** | **ADNI**  **(age: 60-86)** | 60.52 (8.93) | 64.63 (10.86) | 56.06 (10.97) | 60.85 (9.19) | 62.40 (7.10) | 68.44 (8.93) | 56.34 (8.76) | 63.19 (7.64) |
|  |  | **OASIS-3**  **(age: 43-96)** | 61.11 (2.82) | 65.07 (2.74) | 58.70 (3.98) | 61.24 (2.87) | 62.24 (2.64) | 67.37 (3.01) | 59.91 (3.75) | 62.41 (2.70) |
|  |  | **OASIS-3**  **(age: 60-86)** | 61.51 (3.89) | 65.92 (4.09) | 59.82 (4.05) | 61.61 (3.92) | 62.97 (2.77) | 67.95 (3.42) | 61.09 (2.65) | 63.08 (2.85) |
|  | **SVM** | **ADNI**  **(age: 60-86)** | 50.63 (8.00) | 54.42 (11.26) | 49.91 (8.77) | 50.63 (8.05) | 63.65 (6.71) | 70.50 (9.06) | 60.07 (8.16) | 63.87 (6.69) |
|  |  | **OASIS-3**  **(age: 43-96)** | 50.15 (4.10) | 50.38 (5.61) | 47.69 (4.20) | 50.16 (4.14) | 65.13 (2.81) | 70.79 (2.50) | 62.88 (2.90) | 65.28 (2.87) |
|  |  | **OASIS-3**  **(age: 60-86)** | 52.36 (4.18) | 53.44 (6.42) | 51.40 (5.66) | 52.36 (4.20) | 66.58 (2.91) | 72.01 (2.40) | 63.94 (3.41) | 66.78 (2.96) |
|  | **Logistic** | **ADNI**  **(age: 60-86)** | 59.90 (4.32) | 57.83 (8.36) | 64.59 (2.60) | 60.20 (4.28) | 55.52 (8.23) | 60.65 (10.37) | 54.72 (7.80) | 55.59 (8.29) |
|  |  | **OASIS-3**  **(age: 43-96)** | 62.37 (2.13) | 59.08 (2.99) | 66.37 (1.27) | 62.72 (2.06) | 62.44 (3.40) | 66.12 (3.59) | 61.33 (3.78) | 62.47 (3.40) |
|  |  | **OASIS-3**  **(age: 60-86)** | 62.03 (2.59) | 58.17 (3.49) | 66.14 (1.59) | 62.38 (2.53) | 62.08 (5.10) | 66.07 (5.16) | 61.84 (5.04) | 62.09 (5.11) |
|  | **RUSBoost** | **ADNI**  **(age: 60-86)** | 55.31 (10.01) | 53.44 (13.73) | 51.80 (11.19) | 55.53 (10.33) | 56.04 (8.60) | 53.17 (12.20) | 52.14 (12.73) | 56.13 (8.78) |
|  |  | **OASIS-3**  **(age: 43-96)** | 58.61 (3.97) | 60.62 (4.23) | 57.31 (6.98) | 58.72 (4.02) | 58.31 (2.86) | 61.07 (3.46) | 57.77 (3.72) | 58.42 (2.94) |
|  |  | **OASIS-3**  **(age: 60-86)** | 60.80 (4.60) | 62.46 (4.19) | 60.00 (6.98) | 61.08 (4.71) | 60.47 (3.17) | 62.40 (3.50) | 59.12 (6.36) | 60.65 (3.21) |

**Supplementary Table 14:** Comparison of classification performance for imbalanced data analysis for different classification methods and feature selection criteria (see **Figure 5** caption for more information).

| **Features** | **Models** | **Dataset** | **Acc (Mean (SD)) (%)** | **AROC (Mean (SD)) (%)** | **F1**  **(Mean (SD)) (%)** | **MCC (Mean (SD)) (%)** | **Acc (Mean (SD)) (%)** | **AROC (Mean (SD)) (%)** | **F1**  **(Mean (SD)) (%)** | **MCC' (Mean (SD)) (%)** |
| --- | --- | --- | --- | --- | --- | --- | --- | --- | --- | --- |
|  |  |  | **residual harmonization** | | | | **z-score harmonization** | | | |
| 1. **Average score percentage (MCL app): chi-square, ReliefF, ANOVA & Kruskal Wallis** | | | | | | | | | | |
| **brainSegVolNotVent**  **entorhinal**  **inf-Lat-Vent**  **lateral-Ventricle parahippocampal hippocampus**  **temporalpole**  **fusiform**  **lateralorbitofrontal**  **insula**  **precentral**  **isthmuscingulate**  **parsopercularis**  **posteriorcingulate**  **accumbens-area**  **rostralmiddlefrontal**  **middletemporal**  **rostralanteriorcingulate**  **amygdala**  **parsorbitalis** | **Naïve Bayes** | **ADNI**  **(age: 60-86)** | 64.75 (2.31) | 67.72 (3.43) | 36.04 (4.45) | 57.73 (3.09) | 73.93 (3.37) | 68.44 (3.54) | 38.90 (4.76) | 61.34 (3.32) |
|  |  | **OASIS-3**  **(age: 43-96)** | 69.81 (1.73) | 68.03 (0.98) | 37.61 (1.05) | 60.28 (0.81) | 70.34 (0.78) | 68.18 (0.85) | 37.81 (1.38) | 60.50 (0.88) |
|  |  | **OASIS-3**  **(age: 60-86)** | 68.56 (2.48) | 67.22 (1.49) | 40.29 (1.82) | 60.69 (1.47) | 69.82 (0.84) | 67.92 (1.09) | 40.78 (1.18) | 61.19 (0.78) |
|  | **KNN** | **ADNI**  **(age: 60-86)** | 59.09 (3.73) | 63.48 (5.08) | 35.93 (4.90) | 56.96 (3.93) | 61.07 (3.88) | 61.09 (4.62) | 30.47 (3.57) | 53.57 (2.71) |
|  |  | **OASIS-3**  **(age: 43-96)** | 62.82 (1.20) | 67.38 (1.28) | 37.88 (1.30) | 60.04 (1.00) | 65.42 (1.90) | 66.33 (1.71) | 36.13 (1.86) | 58.87 (1.40) |
|  |  | **OASIS-3**  **(age: 60-86)** | 59.77 (2.29) | 64.12 (1.97) | 38.81 (2.30) | 58.75 (1.88) | 66.33 (1.73) | 63.77 (2.27) | 38.59 (2.27) | 59.30 (1.63) |
|  | **SVM** | **ADNI**  **(age: 60-86)** | 80.17  (0) | 2.50 (11.18) | 0  (0) | 0  (0) | 81.90 (1.28) | 64.49 (5.63) | 40.55 (6.09) | 66.80 (2.53) |
|  |  | **OASIS-3**  **(age: 43-96)** | 81.95 (0.22) | 9.10 (22.24) | 1.07 (1.69) | 26.18 (26.93) | 81.60 (0.44) | 60.45 (3.42) | 1.10 (2.07) | 32.32 (24.39) |
|  |  | **OASIS-3**  **(age: 60-86)** | 79.58  (0) | 0  (0) | 0  (0) | 0  (0) | 78.89 (0.52) | 59.22 (2.88) | 3.49 (3.18) | 50.61 (2.19) |
|  | **Logistic** | **ADNI**  **(age: 60-86)** | 51.45 (0.45) | 53.97 (3.98) | 37.06 (0.83) | 57.43 (0.82) | 62.02 (5.97) | 53.10 (5.36) | 26.99 (4.62) | 51.99 (3.02) |
|  |  | **OASIS-3**  **(age: 43-96)** | 54.63 (0.09) | 61.45 (2.79) | 37.11 (0.04) | 59.51 (0.04) | 64.44 (9.19) | 54.38 (2.20) | 24.62 (4.13) | 52.22 (1.80) |
|  |  | **OASIS-3**  **(age: 60-86)** | 55.13 (0.11) | 59.63 (3.46) | 40.39 (0.14) | 59.97 (0.13) | 61.80 (6.78) | 54.53 (2.40) | 27.77 (3.77) | 52.32 (1.95) |
|  | **RUSBoost** | **ADNI**  **(age: 60-86)** | 72.15 (2.57) | 72.20 (3.54) | 39.68 (5.38) | 61.30 (3.41) | 74.09 (2.64) | 74.34 (4.13) | 39.95 (5.74) | 61.89 (3.63) |
|  |  | **OASIS-3**  **(age: 43-96)** | 76.72 (0.81) | 69.73 (1.00) | 39.93 (1.96) | 62.84 (1.21) | 76.15 (0.69) | 69.99 (0.98) | 40.01 (1.64) | 62.74 (1.01) |
|  |  | **OASIS-3**  **(age: 60-86)** | 74.55 (0.85) | 68.67 (1.10) | 40.53 (1.95) | 62.22 (1.18) | 73.82 (1.08) | 68.31 (1.19) | 40.51 (1.88) | 62.10 (1.20) |
| 1. **ReliefF** | | | | | | | | | | |
| **entorhinal**  **fusiform**  **Inf-Lat-Vent**  **Temporalpole**  **Posteriorcingulate**  **Isthmuscingulate**  **hippocampus**  **parahippocampal**  **parsopercularis**  **Lateral-Ventricle**  **transversetemporal**  **precentral**  **insula**  **frontalpole**  **superiortemporal**  **amygdala**  **lateralorbitofrontal**  **lingual**  **BrainSegVolNotVent** | **Naïve Bayes** | **ADNI**  **(age: 60-86)** | 66.40 (2.23) | 69.97 (3.75) | 38.14 (3.39) | 59.32 (2.39) | 71.69 (2.47) | 72.40 (4.35) | 43.26 (4.69) | 63.27 (3.08) |
|  |  | **OASIS-3**  **(age: 43-96)** | 65.21 (1.85) | 66.80 (1.09) | 34.03 (1.55) | 57.46 (1.13) | 72.13 (0.89) | 68.13 (0.78) | 37.00 (1.40) | 60.26 (0.82) |
|  |  | **OASIS-3**  **(age: 60-86)** | 61.57 (2.20) | 66.57 (0.90) | 36.74 (1.22) | 57.41 (1.02) | 71.48 (1.07) | 67.51 (1.02) | 39.46 (1.75) | 60.81 (1.07) |
|  | **KNN** | **ADNI**  **(age: 60-86)** | 60.54 (3.86) | 64.10 (4.18) | 35.11 (4.79) | 56.56 (3.57) | 70.12 (3.80) | 71.52 (3.85) | 41.50 (5.01) | 61.96 (3.51) |
|  |  | **OASIS-3**  **(age: 43-96)** | 61.77 (1.54) | 64.74 (1.61) | 37.57 (1.61) | 59.77 (1.30) | 64.21 (1.63) | 62.66 (1.79) | 34.21 (1.75) | 57.49 (1.13) |
|  |  | **OASIS-3**  **(age: 60-86)** | 60.47 (1.72) | 63.37 (2.00) | 38.85 (2.49) | 58.83 (1.98) | 64.76 (1.72) | 62.90 (1.87) | 37.38 (1.80) | 58.28 (1.33) |
|  | **SVM** | **ADNI**  **(age: 60-86)** | 80.08 (0.37) | 0  (0) | 0  (0) | 2.34 (10.46) | 81.12 (2.25) | 68.08 (4.77) | 38.42 (6.89) | 65.22 (4.20) |
|  |  | **OASIS-3**  **(age: 43-96)** | 82.00 (0.00) | 2.50 (11.18) | 0  (0) | 0  (0) | 81.75 (0.42) | 53.89 (4.01) | 2.90 (2.29) | 52.08 (2.52) |
|  |  | **OASIS-3**  **(age: 60-86)** | 79.58  (0) | 0  (0) | 0  (0) | 0  (0) | 79.36 (0.37) | 55.36 (2.49) | 1.27 (1.66) | 43.27 (18.83) |
|  | **Logistic** | **ADNI**  **(age: 60-86)** | 51.32 (0.37) | 56.12 (4.40) | 36.87 (0.69) | 57.24 (0.68) | 61.69 (8.07) | 56.88 (5.70) | 31.39 (6.98) | 54.46 (5.02) |
|  |  | **OASIS-3**  **(age: 43-96)** | 54.65 (0.10) | 60.38 (2.35) | 37.12 (0.05) | 59.52 (0.04) | 65.64 (8.31) | 53.53 (2.06) | 23.72 (4.45) | 52.12 (1.27) |
|  |  | **OASIS-3**  **(age: 60-86)** | 55.13 (0.15) | 60.63 (2.55) | 40.33 (0.21) | 59.91 (0.20) | 63.53 (7.48) | 55.06 (2.75) | 27.68 (3.49) | 52.85 (2.15) |
|  | **RUSBoost** | **ADNI**  **(age: 60-86)** | 76.03 (2.37) | 80.11 (2.52) | 50.60 (5.20) | 68.31 (3.45) | 77.64 (2.34) | 81.54 (2.92) | 50.44 (5.06) | 68.43 (3.24) |
|  |  | **OASIS-3**  **(age: 43-96)** | 76.52 (0.76) | 69.25 (0.88) | 38.17 (1.89) | 61.90 (1.13) | 75.60 (1.00) | 69.52 (1.08) | 37.85 (1.47) | 61.46 (1.01) |
|  |  | **OASIS-3**  **(age: 60-86)** | 74.56 (1.20) | 67.79 (1.11) | 39.18 (2.48) | 61.57 (1.57) | 73.32 (1.00) | 67.64 (1.37) | 39.45 (1.85) | 61.28 (1.22) |
| 1. **Frequent feature appearances from all feature ranking** **analysis** | | | | | | | | | | |
| **BrainSegVolNotVent**  **Inf-Lat-Vent**  **entorhinal**  **lateralorbitofrontal**  **Lateral-Ventricle**  **parahippocampal**  **hippocampus** | **Naïve Bayes** | **ADNI**  **(age: 60-86)** | 66.94 (3.14) | 71.06 (4.62) | 40.37 (3.95) | 60.80 (2.97) | 77.36 (2.37) | 73.46 (4.40) | 47.48 (3.92) | 66.69 (2.64) |
|  |  | **OASIS-3**  **(age: 43-96)** | 73.63 (1.80) | 69.41 (1.41) | 38.27 (2.03) | 61.24 (1.47) | 72.06 (1.05) | 69.80 (0.75) | 38.08 (1.25) | 60.87 (0.82) |
|  |  | **OASIS-3**  **(age: 60-86)** | 66.91 (2.17) | 68.14 (1.08) | 38.15 (1.64) | 59.12 (1.24) | 72.29 (1.50) | 69.14 (0.94) | 40.64 (1.67) | 61.64 (1.19) |
|  | **KNN** | **ADNI**  **(age: 60-86)** | 63.10 (3.70) | 65.29 (4.04) | 37.93 (4.47) | 58.81 (3.37) | 73.43 (3.05) | 72.70 (3.70) | 47.96 (3.63) | 66.38 (2.54) |
|  |  | **OASIS-3**  **(age: 43-96)** | 66.52 (1.48) | 65.62 (1.96) | 37.00 (1.72) | 59.57 (1.26) | 65.73 (1.59) | 66.90 (1.75) | 36.72 (1.59) | 59.32 (1.15) |
|  |  | **OASIS-3**  **(age: 60-86)** | 62.29 (2.56) | 63.36 (2.23) | 37.64 (2.72) | 58.11 (2.12) | 64.03 (1.83) | 64.10 (1.94) | 37.45 (1.84) | 58.20 (1.41) |
|  | **SVM** | **ADNI**  **(age: 60-86)** | 80.29 (0.90) | 7.30 (17.84) | 4.62 (5.77) | 33.52 (28.45) | 82.93 (1.59) | 75.01 (5.05) | 49.07 (5.99) | 70.21 (3.16) |
|  |  | **OASIS-3**  **(age: 43-96)** | 81.91 (0.17) | 41.68 (24.84) | 2.39 (1.53) | 52.13 (1.81) | 81.97 (1.07) | 56.71 (2.22) | 0.79 (2.43) | 12.87 (22.94) |
|  |  | **OASIS-3**  **(age: 60-86)** | 79.24 (1.14) | 14.42 (2.56) | 3.53 (3.56) | 49.48 (11.94) | 78.95 (0.61) | 58.80 (2.05) | 8.86 (6.37) | 50.84 (12.26) |
|  | **Logistic** | **ADNI**  **(age: 60-86)** | 51.40 (0.69) | 55.10 (4.49) | 37.18 (0.96) | 57.54 (0.94) | 62.85 (6.71) | 56.42 (4.36) | 30.14 (4.73) | 54.11 (3.00) |
|  |  | **OASIS-3**  **(age: 43-96)** | 54.62 (0.06) | 60.72 (2.74) | 37.10 (0.03) | 59.50 (0.03) | 64.34 (9.25) | 53.51 (3.08) | 24.14 (3.39) | 51.84 (2.14) |
|  |  | **OASIS-3**  **(age: 60-86)** | 55.17 (0.17) | 60.05 (2.33) | 40.43 (0.13) | 60.00 (0.12) | 63.23 (6.59) | 54.63 (2.70) | 26.75 (4.02) | 52.20 (2.10) |
|  | **RUSBoost** | **ADNI**  **(age: 60-86)** | 69.83 (2.30) | 66.96 (3.54) | 37.58 (3.92) | 59.56 (2.61) | 70.66 (2.94) | 66.67 (2.93) | 38.05 (3.56) | 59.97 (2.61) |
|  |  | **OASIS-3**  **(age: 43-96)** | 77.36 (0.55) | 70.10 (0.92) | 39.33 (2.05) | 62.74 (1.17) | 76.99 (0.69) | 69.51 (1.27) | 38.11 (1.34) | 62.01 (0.84) |
|  |  | **OASIS-3**  **(age: 60-86)** | 75.69 (1.01) | 67.45 (1.04) | 41.94 (2.36) | 63.31 (1.43) | 75.12 (1.10) | 67.33 (1.07) | 41.26 (2.03) | 62.77 (1.33) |
| 1. **features extracted from statistical analysis (SPSS software): ANOVA, ANCOVA & Kruskal Wallis** | | | | | | | | | | |
| **Entorhinal**  **Inf-Lat-Vent**  **Hippocampus**  **Lateral-Ventricle**  **lateralorbitofrontal**  **BrainSegVolNotVent**  **Accumbens-area**  **middle temporal** | **Naïve Bayes** | **ADNI**  **(age: 60-86)** | 67.15 (1.80) | 71.34 (4.46) | 39.29 (4.37) | 60.21 (2.97) | 77.11 (3.47) | 72.41 (3.50) | 46.97 (4.87) | 66.39 (3.46) |
|  |  | **OASIS-3**  **(age: 43-96)** | 71.81 (1.52) | 69.79 (1.89) | 37.99 (1.49) | 60.78 (1.05) | 71.53 (0.96) | 70.43 (0.83) | 37.43 (1.16) | 60.40 (0.76) |
|  |  | **OASIS-3**  **(age: 60-86)** | 72.24 (1.57) | 70.33 (1.00) | 42.54 (1.71) | 62.67 (1.25) | 70.08 (1.04) | 69.81 (0.91) | 40.73 (1.36) | 61.21 (0.95) |
|  | **KNN** | **ADNI**  **(age: 60-86)** | 60.91 (3.70) | 66.37 (5.05) | 37.15 (4.80) | 58.11 (3.56) | 74.83 (2.89) | 75.50 (3.16) | 47.54 (4.33) | 66.25 (2.97) |
|  |  | **OASIS-3**  **(age: 43-96)** | 67.02 (1.22) | 64.98 (1.96) | 37.94 (1.59) | 60.25 (1.14) | 65.85 (1.58) | 69.20 (1.54) | 39.10 (1.70) | 61.04 (1.23) |
|  |  | **OASIS-3**  **(age: 60-86)** | 65.22 (2.02) | 63.99 (1.67) | 39.09 (1.82) | 59.44 (1.43) | 66.26 (1.73) | 67.11 (1.31) | 41.21 (1.80) | 60.99 (1.35) |
|  | **SVM** | **ADNI**  **(age: 60-86)** | 80.17 (0.54) | 0  (0) | 3.35 (5.61) | 21.89 (27.69) | 82.23 (1.81) | 73.25 (4.02) | 44.77 (6.39) | 68.41 (3.12) |
|  |  | **OASIS-3**  **(age: 43-96)** | 82.04 (0.28) | 39.15 (29.6) | 3.37 (2.19) | 53.40 (2.68) | 81.86 (0.18) | 56.32 (1.90) | 0.08 (0.37) | 24.52 (25.16) |
|  |  | **OASIS-3**  **(age: 60-86)** | 79.58  (0) | 0  (0) | 0  (0) | 0  (0) | 79.35 (0.32) | 55.52 (1.60) | 2.78 (3.53) | 43.66 (19.01) |
|  | **Logistic** | **ADNI**  **(age: 60-86)** | 51.53 (0.49) | 56.59 (5.21) | 37.37 (0.88) | 57.73 (0.86) | 63.43 (6.52) | 57.74 (5.80) | 30.00 (6.99) | 54.23 (4.09) |
|  |  | **OASIS-3**  **(age: 43-96)** | 54.64 (0.08) | 60.83 (2.88) | 37.11 (0.04) | 59.51 (0.03) | 64.19 (11.00) | 53.40 (2.29) | 24.02 (4.25) | 52.21 (1.78) |
|  |  | **OASIS-3**  **(age: 60-86)** | 55.20 (0.16) | 60.06 (2.59) | 40.42 (0.18) | 59.99 (0.17) | 62.95 (7.38) | 54.55 (2.88) | 26.89 (4.84) | 52.21 (2.44) |
|  | **RUSBoost** | **ADNI**  **(age: 60-86)** | 72.02 (1.88) | 69.08 (3.35) | 36.81 (4.93) | 59.66 (3.02) | 74.13 (2.38) | 69.30 (3.15) | 40.42 (5.33) | 62.15 (3.34) |
|  |  | **OASIS-3**  **(age: 43-96)** | 77.03 (0.99) | 67.80 (0.74) | 37.17 (1.95) | 61.57 (1.25) | 76.58 (0.73) | 67.57 (0.61) | 37.08 (1.33) | 61.37 (0.83) |
|  |  | **OASIS-3**  **(age: 60-86)** | 75.14 (1.04) | 66.05 (1.48) | 38.98 (2.63) | 61.70 (1.58) | 74.85 (0.97) | 65.88 (1.18) | 39.47 (2.00) | 61.82 (1.19) |
